# Does body mass index or waist-hip ratio correlate with arterial stiffness based on brachial-ankle pulse wave velocity in Chinese rural adults with hypertension?

**DOI:** 10.1186/s12872-021-02390-y

**Published:** 2021-12-01

**Authors:** Feng Hu, Rihua Yu, Fengyu Han, Juan Li, Wei Zhou, Tao Wang, Lingjuan Zhu, Xiao Huang, Huihui Bao, Xiaoshu Cheng

**Affiliations:** 1 Department of Cardiovascular Medicine, The Second Affiliated Hospital of Nanchang University, No. 1 Minde Road, Nanchang, 330006 Jiangxi China; 2 Jiangxi Provincial Cardiovascular Disease Clinical Medical Research Center, Nanchang, Jiangxi China; 3 Department of General Practice Medicine, Zhentou Town Health Center, Wuyuan, Jiangxi China; 4 The College of Pharmacy, Nanchang University, Nanchang, Jiangxi China; 5 Center for Prevention and Treatment of Cardiovascular Diseases, The Second Affiliated Hospital of Nanchang University, Nanchang, Jiangxi China

**Keywords:** Body mass index, Waist-hip ratio, Arterial stiffness, Brachial-ankle pulse wave velocity, Chinese, Hypertension

## Abstract

**Background:**

The relationship between obesity indices and arterial stiffness (AS) has not been fully discovered nor has it been studied in depth in large hypertensive patient populations. The aim of this study was to explore the association between body mass index (BMI) and waist-hip ratio (WHR) levels and AS based on brachial-ankle pulse wave velocity (baPWV) in Chinese rural adults with hypertension.

**Methods:**

This cross-sectional study analyzed 5049 Chinese rural adults with essential hypertension. BMI was calculated as the body weight in kilograms divided by the square of the height in meters (kg/m^2^). Central obesity was defined as WHR ≥ 0.9 for males and ≥ 0.85 for females. Measurement of arterial stiffness was carried out via brachial-ankle pulse wave velocity (baPWV).

**Results:**

The prevalence of overweight, general obesity, central obesity and increased AS were 26.88%, 3.39%, 63.85% and 44.01%, respectively. Multivariate logistic regression analysis indicated that BMI levels were negatively associated with the prevalence of increased AS (adjusted-OR per *SD* increase: 0.74, 95% *CI* 0.67–0.81, *P* < 0.001). When BMI was instead treated as a categorical variable divided into tertiles, the same relationship was observed (P for trend < 0.001). Inversely, WHR levels were positively associated with the prevalence of increased AS (adjusted-OR per *SD* increase: 1.25, 95% CI 1.14–1.36, P < 0.001). Compared to subjects without central obesity, those with central obesity had a higher prevalence of increased AS (adjusted-OR: 1.52, 95% *CI* 1.28–1.81, P < 0.001). Linear regression models indicated similar results in the correlation between BMI or WHR levels and baPWV levels (adjusted-*β* per *SD* increase: − 0.57, 95% *CI* − 0.68 to − 0.46, *P* < 0.001; adjusted-*β* per *SD* increase: 4.46, 95% *CI* 3.04–5.88, *P* < 0.001). There were no interactions in terms of age and blood pressure on the relationship between BMI or WHR levels and the prevalence of increased AS or baPWV levels.

**Conclusion:**

There was an inverse relationship between BMI levels and increased AS or baPWV levels, whereas WHR levels and central obesity were positively associated with increased AS or baPWV levels in Chinese rural adults with hypertension.

**Supplementary Information:**

The online version contains supplementary material available at 10.1186/s12872-021-02390-y.

## Background

Cardiovascular diseases (CVDs) constitute the leading cause of global mortality including China [1]. The prevalence of global obesity has steadily increased in the past few decades and has increased the risk of CVDs [2]. Approximately 23.2% of adults in China are estimated to suffer from hypertension, and hypertension is often accompanied by obesity [3]. Arterial stiffness (AS) has a crucial role in the pathogenesis of hypertension, which could prognosticate the risk rate of subclinical vascular disorders and cardiovascular events in hypertensive patients [4]. Increased AS represents a valuable metric to guide the stratification of hypertensive patients based on their cardiovascular risk [5].

Carotid-femoral pulse wave velocity (cfPWV) is currently considered as the gold standard methodology for evaluating AS [6], whereas brachial-ankle pulse wave velocity (baPWV) is equally regard as a risk biomarker of CVDs and is strongly linked to aortic PWV and cfPWV [7]. In the same way, baPWV has been accepted as an effective and repeatable metric tool to assessing AS in many epidemiological surveys investigation [8–13]. Therefore, baPWV was adopted as a metric for AS in this study.

Body mass index (BMI) is one of the main measures for general obesity and is commonly used in clinical practice. However, research on the association between BMI and PWV in various populations, including hypertensive patients [14–19], remains controversial. Studies have reported an insignificant relationship [14, 18, 20–24], positive relationship [15, 17, 19, 25–28], or negative relationship [16, 29–33]. Many causes could interpret these apparent discrepancies including diverse sample size, different origins of the study population, ethnic and regional disparity. On the other hand, BMI is not able to differentiate fat-free muscle from fat mass. Furthermore, BMI cannot account for sex and racial differences in fat content, and it is not able to differentiate the distribution of visceral and subcutaneous fat [34]. Thus, more studies need to verify the relationship between the BMI levels and AS based on PWV.

Waist-hip ratio (WHR), a metric of central obesity less influenced by muscle and bone mass, might be better indicate risk for CVDs associated with obesity than BMI [35, 36]. Higher WHR was significantly correlated with increased AS based on PWV [19, 25, 27]. This study was designed for evaluating the relationship between the BMI or WHR levels and the prevalence of increased AS and baPWV in a large real-world population of Chinese hypertensive rural adults.

## Methods

### Participants

All subjects in the present study were participants in the ongoing China H-type hypertension Registry Study (Registration number: ChiCTR1800017274).The data collection approaches and the established standards of inclusion or exclusion have been described previously [37, 38]. Briefly, the study is a real-world, multicenter, observational study, conducted in March 2018 at Wuyuan, Jiangxi province of China. Enrolled population were hypertensive patients who aged 18 years and older. The exclusion criteria included psychological or nervous system impairment resulting in an inability to demonstrate informed consent, unable to be long-term followed-up according to the study protocol, or plans to relocate in the near future.

Of the eligible patients, 5, 233 completed baPWV measurements. After excluding 2 participants without BMI measurements, 78 individuals with ankle brachial index (ABI) < 0.9 [11, 24] and 104 patients with atrial fibrillation, finally 5, 049 participants were included in our analysis (Additional file 2: Fig. 1).

### Clinical data collection

Participants’ demographic characteristics (age, gender), lifestyle factors (smoking and drinking status), medical history [diabetes mellitus (DM), coronary heart disease (CHD), stroke, dyslipidemia and atrial fibrillation] and medication usage were gathered by professional researchers through questionnaire survey. Atrial fibrillation was diagnosed based on a medical history and through resting supine standard 12-lead surface electrocardiogram.

Anthropometric measurements for each patient including weight, height, waistline, hipline, systolic blood pressure (SBP), diastolic blood pressure (DBP) and heart rate were obtained by researchers. Waistline and hipline were measured using an inelastic measuring tape Blood pressure (BP) was measured, with the participant in a sitting position using the Omron HBP-1300 Professional Portable Blood Pressure Monitor (Kyoto, Japan) on the right arm, which was supported at the heart level. After a 5-min rest period, BP was measured four times, and SBP and DBP were calculated as the average of the last three readings. BMI was calculated as the body weight in kilograms divided by the square of the height in meters (kg/m^2^). WHR was calculated as the waistline in centimeters divided by the hipline in centimeters. In our study, subjects were divided into three categories according to BMI levels (< 25 kg/m^^2^, control group; 25‐30 kg/m^^2^, overweight; and ≥ 30 kg/m^^2^, general obesity) [25–27, 30]. Central obesity was defined as WHR ≥ 0.9 for males and ≥ 0.85 for females [39].

### Laboratory assay

Blood samples were collected utilizing venipuncture after an overnight fast of at least 12 h. The levels of plasma total homocysteine, fasting blood glucose (FBG), total cholesterol (TC), total triglyceride (TG), high-density lipoprotein cholesterol (HDL-C), low-density lipoprotein cholesterol (LDL-C), serum uric acid and creatinine, blood urea nitrogen (BUN), total and direct bilirubin, aspartate aminotransferase (AST) and alanine aminotransferase (ALT) were measured using automatic clinical analyzers (Beckman Coulter) In our analysis, diagnosis of incident diabetes was defined as fasting glucose > 7.0 mmol/l, and/or self-reported diabetes.. Dyslipidemia was defined as having one or more of the following features: elevated TG (≥ 2.3 mmol/L), elevated TC (≥ 6.2 mmol/L), elevated LDL-C (≥ 4.1 mmol/L) and reduced HDL-C (< 1.0 mmol/L) or on appropriate lipid-lowering medication..The estimated glomerular filtration rate (eGFR) was calculated by the equation of Chronic Kidney Disease Epidemiology Collaboration (CKD-EPI).

### Measurement of baPWV and definition of arterial stiffness

BaPWV and the ABI were measured using a BP-203RPEIII networked arteriosclerosis detection device (Omron Health Care, Kyoto, Japan). Participants received this measurement after at least 5 min of rest in the supine position in a quiet room. The measurement approaches of baPWV and ABI related to this study have been described previously [37, 38]. The average of baPWV measured on bilateral limbs of each patient was used for analysis. While baPWV value has been shown to have prognostic significance, with a threshold of increased risk that exist around 18 m/s [11]. Therefore, baPWV ≥ 18 m/s was considered as increased AS in our analysis.

### Statistical analysis

Continuous variables are presented as the means ± standard deviation (SD) or the median (quartiles), as appropriate, and are compared using the Student's t test, one-way analysis or the Mann Whitney *U* test, depending on whether the quantitative data were consistent with a normal distribution. Categorical variables were presented as count (percentage), differences between groups were measured by chi-square test.

Multivariate logistic regression models based on odds ratio (OR) with their associated 95% confidence interval (CI) were used to estimate the association of BMI or WHR levels with increased AS. Linear regression models indicated were used to estimate the effect size (*β*) of BMI or WHR levels on baPWV levels. The crude model was not adjusted for any confounder. The model I was adjusted for age, gender, SBP, DBP, heart rate, WHR or BMI. The model II was confounder model. The confounder model screened covariates including age, sex, SBP, DBP, heart rate, BMI, WHR, DM, CHD, history of stroke, smoking and drinking status, total homocysteine, TC, TG, HDL-C, LDL-C, AST, ALT, serum uric acid, eGFR, total and direct bilirubin, antihypertensive medications, lipid-lowering agents and antiplatelet agents except the independent variable itself. We selected these confounders on the basis that, when added to this model, it changed the matched odds ratio by at least 10 percent. Additional file 1: Table 1 showed the association of each confounder with the outcomes of interest. We considered the confounder model to be the main model. In addition, we performed tests for linear trend by entering the median value of each category of BMI or WHR levels as a continuous variable in the models.

Furthermore, the generalized additive model and smooth curve fitting (penalized spline method) was used to visually show the relationship of BMI or WHR levels with baPWV levels. Subgroup analyses were conducted with a stratified multivariate regression approach, and interaction analyses were presented in tabulated form.

All statistical analyses were performed using the statistical package R (http://www.R-project.org, The R Foundation, version 3.4.3) and the Empower (R; www.empowerstats.com; X&Y Solutions, Inc, Boston, MA, USA). All P-values are two-tailed, and P < 0.05 was considered statistically significant.

## Results

### Clinical characteristics of study population

The present study included 5, 049 Chinese adult hypertensive individuals (mean age: 64.46 ± 9.45 years, range 29–93 years; male, 49.89%), and the prevalence of overweight, general obesity, central obesity and increased AS were 26.88%, 3.39%, 63.85% and 44.01%, respectively. The clinical characteristics of the study participants grouped by BMI tertiles or central obesity were presented in Table 1. Compared to BMI ≤ 21.68 kg/m^^2^, there were lower age and SBP, smaller baPWV levels s, a lower prevalence of increased AS, greater DBP and WHR, a higher prevalence of central obesity for the participants in the second and highest tertile of BMI (all *P* values < 0.05, Table 1). Compared to subjects without central obesity, those with central obesity had lower age, greater BMI and baPWV levels, a higher prevalence of increased AS and overweight as well as general obesity (all *P* values < 0.05, Table 1). In this study, 184 patients with diabetes, CHD or stroke were treated with antiplatelet drugs and thus, further adjustments for these conditions were performed in different regression models.

**Table 1:** Clinical characteristics of participants grouped by BMI tertiles or central obesity

Characteristics	BMI tertiles (kg/m^^2^)			*P-value*	Central obesity		*P-value*
	T1 [13.83, 21.68]	T2 [21.68, 24.69]	T3 [24.69, 46.43]		No	Yes	
*Number of subjects (n)*	1683	1683	1683		1825	3224	
Age (years)	68.13 ± 8.84	63.89 ± 8.72	61.35 ± 9.51	< 0.001	64.98 ± 9.61	64.16 ± 9.35	0.004
Male, n (%)	898 (53.36%)	820 (48.72%)	801 (47.59%)	0.002	1183 (64.82%)	1336 (41.44%)	< 0.001
SBP (mmHg)	147.81 ± 18.37	147.00 ± 17.72	146.13 ± 16.49	0.021	147.05 ± 18.26	146.94 ± 17.15	0.845
DBP (mmHg)	86.30 ± 11.24	88.99 ± 10.51	90.82 ± 10.55	< 0.001	88.24 ± 11.14	88.97 ± 10.80	0.054
HR (times/min)	75.40 ± 15.56	74.98 ± 13.50	76.89 ± 13.91	< 0.001	74.16 ± 14.44	76.66 ± 14.26	< 0.001
Height (cm)	155.22 ± 8.11	155.90 ± 8.09	156.51 ± 8.27	< 0.001	157.35 ± 7.72	155.05 ± 8.30	< 0.001
Weigh (kg)	47.41 ± 6.25	56.58 ± 6.30	66.62 ± 8.60	< 0.001	52.29 ± 8.85	59.46 ± 10.64	< 0.001
BMI (kg/m^^2^)	19.62 ± 1.53	23.21 ± 0.86	27.14 ± 2.27	< 0.001	21.04 ± 2.70	24.61 ± 3.21	< 0.001
BMI category (kg/m^^2^)				< 0.001			< 0.001
Control (< 25)	1683 (100.00%)	1683 (100.00%)	155 (9.21%)		1684 (92.27%)	1837 (56.98%)	
Overweight (≥ 25, < 30)	0 (0.00%)	0 (0.00%)	1357 (80.63%)		135 (7.40%)	1222 (37.90%)	
General obesity (≥ 30)	0 (0.00%)	0 (0.00%)	171 (10.16%)		6 (0.33%)	165 (5.12%)	
Waistline (cm)	73.29 ± 6.38	82.39 ± 5.46	91.27 ± 6.47	< 0.001			
Hipline (cm)	86.03 ± 4.41	91.39 ± 4.54	97.05 ± 5.94	< 0.001	89.28 ± 6.46	92.74 ± 6.57	< 0.001
WHR	0.85 ± 0.06	0.90 ± 0.06	0.94 ± 0.06	< 0.001	0.83 ± 0.04	0.94 ± 0.05	< 0.001
Central obesity, n (%)	565 (33.57%)	1143 (67.91%)	1516 (90.08%)	< 0.001			
baPWV (m/s)	18.97 ± 4.34	17.92 ± 3.63	17.35 ± 3.42	< 0.001	17.94 ± 3.85	18.16 ± 3.89	0.010
Increased AS, n (%)	877 (52.11%)	715 (42.48%)	630 (37.43%)	< 0.001	743 (40.71%)	1479 (45.87%)	< 0.001
Smoking status, n (%)				< 0.001			< 0.001
Never	771 (45.81%)	930 (55.26%)	990 (58.82%)		766 (41.97%)	1925 (59.71%)	
Former smoker	292 (17.35%)	317 (18.84%)	323 (19.19%)		373 (20.44%)	559 (17.34%)	
Current smoker	620 (36.84%)	436 (25.91%)	370 (21.98%)		686 (37.59%)	740 (22.95%)	
Drinking status, n (%)				0.076			< 0.001
Never	1030 (61.20%)	1060 (62.98%)	1104 (65.60%)		1043 (57.15%)	2151 (66.72%)	
Former drinker	219 (13.01%)	211 (12.54%)	179 (10.64%)		259 (14.19%)	350 (10.86%)	
Current drinker	434 (25.79%)	412 (24.48%)	400 (23.77%)		523 (28.66%)	723 (22.43%)	
Homocysteine (μmol/L)	15.75 (12.91–20.88)	14.87 (12.40–19.20)	14.93 (12.46–18.76)	< 0.001	15.62 (12.79–21.14)	14.91 (12.50–19.02)	< 0.001
FBG (mmol/L)	5.80 ± 1.11	6.18 ± 1.63	6.44 ± 1.97	< 0.001	5.80 ± 1.10	6.33 ± 1.84	< 0.001
TC (mmol/L)	4.99 ± 1.07	5.13 ± 1.14	5.25 ± 1.12	< 0.001	4.96 ± 1.07	5.22 ± 1.13	< 0.001
TG (mmol/L)	1.28 ± 0.71	1.81 ± 1.28	2.18 ± 1.52	< 0.001	1.12 (0.83–1.56)	1.63 (1.16–2.35)	< 0.001
HDL-C (mmol/L)	1.63 ± 0.43	1.47 ± 0.38	1.38 ± 0.34	< 0.001	1.60 ± 0.43	1.43 ± 0.36	< 0.001
LDL-C (mmol/L)	2.71 ± 0.74	2.94 ± 0.80	3.12 ± 0.79	< 0.001	2.71 ± 0.74	3.04 ± 0.80	< 0.001
Serum uric acid (mmol/L)	414.96 ± 117.95	425.37 ± 117.41	454.13 ± 125.18	< 0.001	422.65 ± 116.69	436.49 ± 123.63	< 0.001
Serum creatinine (mmol/L)	68.00 (57.00–84.00)	67.00 (55.00–81.00)	68.00 (57.00–84.00)	0.046	71.00 (59.00–86.00)	66.00 (55.00–81.00)	< 0.001
BUN (mmol/L)	5.65 ± 2.07	5.37 ± 1.69	5.30 ± 1.70	< 0.001	5.58 ± 2.05	5.36 ± 1.70	0.002
eGFR (ml/min/1.73m^2^)	83.19 ± 19.96	87.36 ± 18.69	87.62 ± 19.87	< 0.001	85.33 ± 20.32	86.47 ± 19.19	0.120
Total bilirubin (mmol/L)	14.21 ± 6.59	14.23 ± 6.06	14.62 ± 6.35	0.107	14.42 ± 6.24	14.31 ± 6.40	0.306
Direct bilirubin (mmol/L)	5.45 ± 2.23	5.28 ± 1.96	5.35 ± 1.95	0.269	5.54 ± 2.13	5.26 ± 2.00	< 0.001
AST (U/L)	24.00 (20.00–30.00)	24.00 (20.00–29.00)	24.00 (20.00–31.00)	0.003	24.00 (20.00–30.00)	24.00 (20.00–30.00)	0.212
ALT (U/L)	15.00 (11.00–19.00)	17.00 (13.00–23.00)	21.00 (15.00–29.00)	< 0.001	15.00 (12.00–21.00)	18.00 (13.00–26.00)	< 0.001
DM, n (%)	199 (11.82%)	323 (19.19%)	413 (24.54%)	< 0.001	199 (10.90%)	736 (22.83%)	< 0.001
CHD, n (%)	116 (6.89%)	104 (6.18%)	114 (6.77%)	0.672	111 (6.08%)	223 (6.92%)	
History of stroke, n (%)	130 (7.72%)	132 (7.84%)	113 (6.71%)	0.390	137 (7.51%)	238 (7.38%)	
Dyslipidemia, n (%)	364 (21.63%)	669 (39.75%)	818 (48.60%)	< 0.001	440 (24.11%)	1411 (43.77%)	< 0.001
Antihypertensive medications, n (%)	938 (55.73%)	1056 (62.75%)	1084 (64.41%)	< 0.001	1037 (56.82%)	2041 (63.31%)	< 0.001
Hypoglycemic agents, n (%)	39 (2.32%)	84 (4.99%)	101 (6.00%)	< 0.001	30 (1.64%)	194 (6.02%)	< 0.001
Lipid-lowering agents, n (%)	33 (1.96%)	62 (3.68%)	77 (4.58%)	< 0.001	47 (2.58%)	125 (3.88%)	0.014
Antiplatelet agents, n (%)	43 (2.55%)	65 (3.86%)	76 (4.52%)	0.008	58 (3.18%)	126 (3.91%)	0.184

BMI, body mass index; SBP, systolic blood pressure; DBP, diastolic blood pressure; HR, heart rate; WHR, waist hip rate; baPWV, brachial-ankle pulse wave velocity; AS, arterial stiffness; FBG, fasting blood glucose; TC, total cholesterol; TG, total triglyceride; HDL-C, high-density lipoprotein cholesterol; LDL-C, low-density lipoprotein cholesterol; BUN, blood urea nitrogen; eGFR, estimated glomerular filtration rate; AST, aspartate aminotransferase; ALT, alanine aminotransferase; DM, diabetes mellitus; CHD, coronary heart disease

There was a significantly positive correlation between the BMI levels and the WHR levels (*r* = 0.55, *P* < 0.001, Additional file 3: Fig. 2). Linear regression models indicated that BMI levels were positively associated with WHR levels (*β* per *SD* increase: 0.04, 95% *CI* 0.04–0.04, *P* < 0.001; Additional file 1: Table 2). Multivariate logistic regression analysis suggested that BMI levels were positively associated with the prevalence of central obesity (adjusted-OR per *SD* increase: 4.41, 95% *CI* 4.02–4.84, *P* < 0.001; Additional file 1: Table 2). Compared to BMI ≤ 21.68 kg/m^^2^, there were greater WHR values and a higher prevalence of central obesity for the participants in the second and highest tertile of BMI (*β*: 0.05, 95% *CI* 0.05–0.05, *P* < 0.001; *β*: 0.09, 95% *CI* 0.09–0.09, *P* < 0.001, respectively; *P* for trend < 0.001. OR: 4.19, 95% *CI* 3.63–4.84, *P* < 0.001; OR: 17.96, 95% *CI* 14.87–21.70, *P* < 0.001, respectively; *P* for trend < 0.001; Additional file 1: Table 2).

Clinical characteristics of participants grouped by baPWV quartiles were also presented in Additional file 1: Table 3. Compared to baPWV ≤ 15.36 m/s, there were reduced eGFR and BMI levels, a lower prevalence of overweight or general obesity, greater extents of age, SBP and DBP, an higher prevalence of central obesity in the third and highest baPWV quartiles (all *P* values < 0.05, Additional file 1: Table 3).

### Association between BMI levels and the prevalence of increased arterial stiffness

Multivariate logistic regression analysis indicated that BMI levels were negatively associated with the prevalence of increased AS (adjusted-OR per *SD* increase: 0.74, 95% *CI* 0.67–0.81, *P* < 0.001; Table 2). Compared to control group, patients with overweight and general obesity had a lower prevalence of increased AS (adjusted-OR: 0.73, 95% *CI* 0.61–0.87, *P* < 0.001; adjusted-OR: 0.51, 95% *CI* 0.34–0.78, *P* = 0.002, respectively; *P* for trend < 0.001; Table 2). Compared to BMI ≤ 21.68 kg/m^^2^, there were a lower prevalence of increased AS for the participants in the second and highest tertile of BMI (adjusted-OR: 0.71, 95% *CI* 0.59–0.86, *P* < 0.001; adjusted-OR: 0.56, 95% *CI* 0.45–0.69, *P* < 0.001, respectively; *P* for trend < 0.001; Table 2).

**Table 2:** Relationship between BMI levels and the prevalence of increased arterial stiffness in different models

Variables	Event, n (%)	Crude Model		Model I		Model II	
		OR (95%CI)	*P-value*	OR (95%CI)	*P-value*	OR (95%CI)	*P-value*
BMI (kg/m^^2^)							
Per *SD* increase	2222 (44.01%)	0.77 (0.73, 0.82)	< 0.001	0.80 (0.74, 0.88)	< 0.001	0.74 (0.67, 0.81)	< 0.001
BMI category (kg/m^^2^)							
Control (< 25)	1653 (46.95%)	*Ref*		*Ref*		*Ref*	
Overweight (≥ 25, < 30)	514 (37.88%)	0.69 (0.61, 0.78)	< 0.001	0.80 (0.67, 0.95)	< 0.001	0.73 (0.61, 0.87)	< 0.001
General obesity (≥ 30)	55 (32.16%)	0.54 (0.39, 0.74)	< 0.001	0.56 (0.37, 0.84)	0.005	0.51 (0.34, 0.78)	0.002
*P-value* for trend		< 0.001		< 0.001		< 0.001	
BMI tertiles (kg/m^^2^)							
T1 [13.83, 21.68]	877 (52.11%)	*Ref*		*Ref*		*Ref*	
T2 [21.68, 24.69]	715 (42.48%)	0.68 (0.59, 0.78)	< 0.001	0.81 (0.67, 0.96)	0.018	0.71 (0.59, 0.86)	< 0.001
T3 [24.69, 46.43]	630 (37.43%)	0.55 (0.48, 0.63)	< 0.001	0.67 (0.54, 0.82)	< 0.001	0.56 (0.45, 0.69)	< 0.001
*P-value* for trend		< 0.001		< 0.001		< 0.001	

BMI, body mass index; *Ref*, reference; OR, odds ratio; CI, confidence interval; *SD*, standard deviation

Model I adjusted for age, sex, SBP, DBP, HR and WHR

Model II adjusted for age, sex, SBP, DBP, HR, WHR, smoking status, TG, LDL-C, eGFR, DM, CHD, history of stroke and antihypertensive medications

In consideration of that increased AS is closely related to age and BP [11–13, 32, 40, 41], stratified and interaction analyses we ceronducted to explore the effects of age, SBP, and DBP on the association between BMI levels and the prevalence of increased AS. Subgroup analyses indicated that the inverse relationship between BMI levels and the prevalence of increased AS was still stable in the tertiles of age stratification no matter what using BMI levels as continuous variable or the lower tertile of BMI as reference variable (*P-value* for interaction was 0.770 in the main confounder model, Additional file 1: Table 4). Likewise, subgroup analyses also indicated that the reverse relationship between BMI levels and the prevalence of increased AS was still stable in the tertiles of SBP or DBP stratification no matter what using BMI levels as continuous variable or the lower tertile of BMI as reference variable (*P-value* for interaction respectively were 0.566 and 352 in the main confounder model, Additional file 1: Table 5 and 6).

### Association between WHR levels and the prevalence of increased arterial stiffness

Multivariate logistic regression analysis indicated that WHR levels were positively associated with the prevalence of increased AS (adjusted-OR per *SD* increase: 1.25, 95% *CI* 1.14–1.36, *P* < 0.001; Table 3). Compared to subjects without central obesity, those with central obesity had a higher prevalence of increased AS (adjusted-OR: 1.52, 95% *CI* 1.28–1.81, *P* < 0.001; Table 3). Compared to WHR ≤ 0.87, there were a higher prevalence of increased AS for the participants in the second and highest tertile of WHR (adjusted-OR: 1.36, 95% *CI* 1.14–1.64, *P* < 0.001; adjusted-OR: 1.81, 95% *CI* 1.47–2.22, *P* < 0.001, respectively; *P* for trend < 0.001; Table 3).

**Table 3:** Relationship between WHR levels and the prevalence of increased arterial stiffness

Variables	Event, n (%)	Crude Model		Model I		Model II	
		OR (95%CI)	*P-value*	OR (95%CI)	*P-value*	OR (95%CI)	*P-value*
WHR							
Per *SD* increase	2222 (44.01%)	1.11 (1.05, 1.18)	< 0.001	1.28 (1.18, 1.40)	< 0.001	1.25 (1.14, 1.36)	< 0.001
Central obesity							
No	743 (40.71%)	*Ref*		*Ref*		*Ref*	
Yes	1479 (45.87%)	1.23 (1.10, 1.39)	< 0.001	1.62 (1.36, 1.92)	< 0.001	1.52 (1.28, 1.81)	< 0.001
WHR tertiles							
T1 [0.53, 0.87]	697 (41.41%)	*Ref*		*Ref*		*Ref*	
T2 [0.87, 0.93]	716 (42.70%)	1.05 (0.92, 1.21)	0.452	1.41 (1.18, 1.69)	< 0.001	1.36 (1.14, 1.64)	< 0.001
T3 [0.93, 1.68]	809 (47.90%)	1.30 (1.14, 1.49)	< 0.001	1.93 (1.57, 2.36)	< 0.001	1.81 (1.47, 2.22)	< 0.001
P for trend		< 0.001		< 0.001		< 0.001	

WHR, waist hip rate; *Ref*, reference; OR, odds ratio; CI, confidence interval; *SD*, standard deviation

Model I adjusted for age, sex, SBP, DBP, HR and BMI

Model II adjusted for age, sex, SBP, DBP, HR, BMI, smoking status, ALT, HDL-C, eGFR, DM, CHD, history of stroke and antihypertensive medications

Stratified and interaction analyses indicated that the positive relationship between WHR levels and the prevalence of increased AS was still stable in the tertiles of age, SBP or DBP stratification no matter what using the WHR levels as continuous variable or the lower tertile of BMI as reference variable (all *P-value* for interaction respectively were greater than 0.05 in the main confounder model, Additional file 1: Table 7, 8 and 9). Likewise, subgroup analyses also indicated that the positive relationship between central obesity and the prevalence of increased AS were still stable in the tertiles of age, SBP or DBP stratification compared to those without central obesity (all *P-value* for interaction respectively were greater than 0.05 in the main confounder model, Additional file 1: Table 7, 8 and 9).

### Relationship between the BMI levels and baPWV levels

**Fig. 1:**
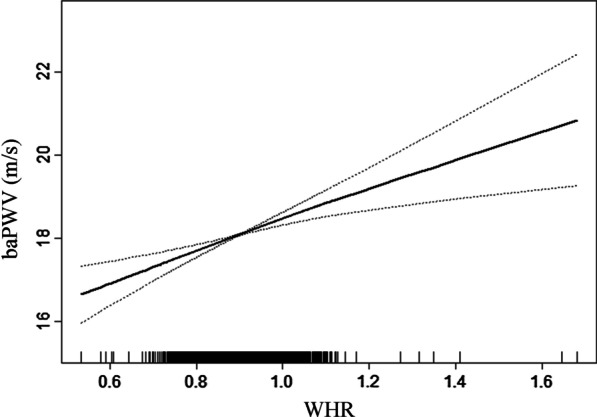
Smooth curve of correlation between BMI levels and baPWV levels. BMI, Body mass index; baPWV, brachial-ankle pulse wave velocity. Smooth curve adjusted for age, sex, SBP, DBP, HR, WHR, eGFR, DM, CHD, history of stroke and antihypertensive medications.

Linear regression models indicated that BMI levels were negatively associated with baPWV levels (adjusted-*β* per *SD* increase: − 0.57, 95% *CI* − 0.68 to − 0.46, *P* < 0.001; Table 4). Compared to control group, patients with overweight and general obesity had lower baPWV levels (adjusted-*β*: − 0.58, 95% *CI* − 0.79 to − 0.37, *P* < 0.001; *β*: − 1.20, 95% *CI* − 1.67 to − 0.72, *P* < 0.001, respectively; *P* for trend < 0.001; Table 4). Compared to BMI ≤ 21.68 kg/m^^2^, there were lower baPWV levels in the second and highest tertile of BMI (adjusted-*β*: − 0.61, 95% *CI* − 0.83 to − 0.40, *P* < 0.001; *β*: − 1.06, 95% *CI* − 1.31 to − 0.81, *P* < 0.001, respectively; *P* for trend < 0.001; Table 4). The smooth curve fitting further confirmed this reverse relationship between BMI levels and baPWV levels (Fig. 1).

**Table 4:** Relationship between BMI levels and baPWV levels in different models

Variables	Crude Model		Model I		Model II	
	*β* (95%CI)	*P-value*	*β* (95%CI)	*P-value*	*β* (95%CI)	*P-value*
BMI (kg/m^^2^)						
Per *SD* increase	− 0.72 (− 0.82, − 0.61)	< 0.001	− 0.52 (− 0.63, − 0.42)	< 0.001	− 0.57 (− 0.68, − 0.46)	< 0.001
BMI category (kg/m^^2^)						
Control (< 25)	*Ref*		*Ref*		*Ref*	
Overweight (≥ 25, < 30)	− 1.02 (− 1.26, − 0.78)	< 0.001	− 0.53 (− 0.74, − 0.33)	< 0.001	− 0.58 (− 0.79, − 0.37)	< 0.001
General obesity (≥ 30)	− 1.67 (− 2.26, − 1.08)	< 0.001	− 1.14 (− 1.61, − 0.66)	< 0.001	− 1.20 (− 1.67, − 0.72)	< 0.001
*P-value* for trend	< 0.001		< 0.001		< 0.001	
BMI tertiles (kg/m^^2^)						
T1 [13.83, 21.68]	*Ref*		*Ref*		*Ref*	
T2 [21.68, 24.69]	− 1.06 (− 1.31, − 0.80)	< 0.001	− 0.55 (− 0.77, − 0.33)	< 0.001	− 0.61 (− 0.83, − 0.40)	< 0.001
T3 [24.69, 46.43]	− 1.62 (− 1.88, − 1.37)	< 0.001	− 0.96 (− 1.21, − 0.71)	< 0.001	− 1.06 (− 1.31, − 0.81)	< 0.001
*P-value* for trend	< 0.001		< 0.001		< 0.001	

BMI, body mass index; baPWV, brachial-ankle pulse wave velocity; *Ref*, reference; *β*, effect size; CI, confidence interval; *SD*, standard deviation

Model I adjusted for age, sex, SBP, DBP, HR and WHR

Model II adjusted for age, sex, SBP, DBP, HR, WHR, eGFR, DM, CHD, history of stroke and antihypertensive medications

To explore whether the negative correlation between BMI tertiles and baPWV levels were still stable in different subgroups, we conducted stratified and interaction analyses. There were not significant interactions in any of the following subgroups, including age tertiles, sex (male vs. female), SBP (< 140 vs. ≥ 140 mmHg), DBP (< 90 vs. ≥ 90 mmHg), central obesity (no vs. yes), DM (no vs. yes), eGFR (≥ 60 vs. < 60 ml/min/1.73 m^^2^) and antihypertensive medication (no vs. yes) (all *P-value* for interaction respectively were greater than 0.05; Additional file 1: Table 10).

### Relationship between the WHR levels and baPWV levels

**Fig. 2:**
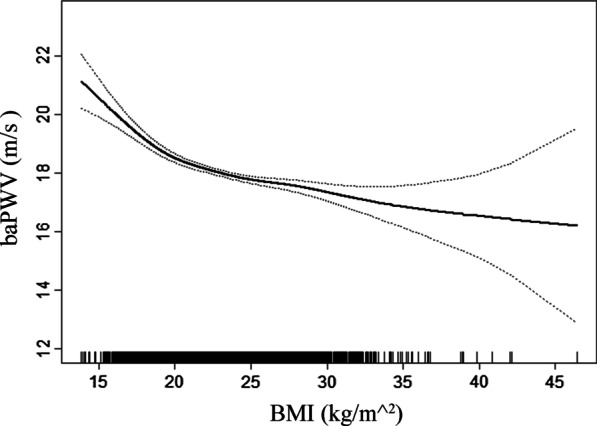
Smooth curve of correlation between WHR levels and baPWV levels. WHR, waist hip rate; baPWV, brachial-ankle pulse wave velocity. Smooth curve adjusted for age, sex, SBP, DBP, HR, WHR, smoking and drinking status, homocysteine, ALT, eGFR, HDL-C, DM, CHD, history of stroke and antihypertensive medications

Linear regression models indicated that WHR levels were positively associated with baPWV levels (adjusted-*β* per *SD* increase: 4.46, 95% *CI* 3.04–5.88, *P* < 0.001; Table 5). Compared to subjects without central obesity, those with central obesity had greater baPWV levels (adjusted-*β*: 0.54, 95% *CI* 0.33–0.74, *P* < 0.001; Table 5). Compared to WHR ≤ 0.87, there were greater baPWV levels in the second and highest tertile of BMI (adjusted-*β*: 0.49, 95% *CI* 0.28–0.70, *P* < 0.001; *β*: 0.80, 95% *CI* 0.56–1.04, *P* < 0.001, respectively; *P* for trend < 0.001; Table 5). The smooth curve fitting further confirmed this positive relationship between WHR levels and baPWV levels (Fig. 2).

**Table 5:** Relationship between WHR levels and baPWV levels in different models

Variables	Crude Model		Model I		Model II	
	*β* (95%CI)	*P-value*	*β* (95%CI)	*P-value*	*β* (95%CI)	*P-value*
WHR						
Per *SD* increase	1.72 (0.21, 3.22)	0.026	5.04 (3.63, 6.45)	< 0.001	4.46 (3.04, 5.88)	< 0.001
Central obesity						
No	*Ref*		*Ref*		*Ref*	
Yes	0.22 (− 0.00, 0.44)	0.052	0.62 (0.42, 0.82)	< 0.001	0.54 (0.33, 0.74)	< 0.001
WHR tertiles						
T1 [0.53, 0.87]	*Ref*		*Ref*		*Ref*	
T2 [0.87, 0.93]	− 0.00 (− 0.26, 0.26)	0.995	0.54 (0.32, 0.75)	< 0.001	0.49 (0.28, 0.70)	< 0.001
T3 [0.93, 1.65]	0.27 (0.01, 0.53)	0.041	0.90 (0.66, 1.14)	< 0.001	0.80 (0.56, 1.04)	< 0.001
*P-value* for trend	< 0.001		< 0.001		< 0.001	

WHR, waist hip rate; baPWV, brachial-ankle pulse wave velocity; *Ref*, reference; *β*, effect size; CI, confidence interval; *SD*, standard deviation

Model I adjusted for age, sex, SBP, DBP, HR and BMI

Model II adjusted for age, sex, SBP, DBP, HR, WHR, smoking and drinking status, homocysteine, ALT, eGFR, HDL-C, DM, CHD, history of stroke and antihypertensive medications

To explore whether the positive correlation between WHR levels and baPWV levels were still stable in different subgroups, we also conducted stratified and interaction analyses. There were not significant interactions in any of the following subgroups, including age tertiles, sex (male vs. female), SBP (< 140 vs. ≥ 140 mmHg), DBP (< 90 vs. ≥ 90 mmHg), smoking habit (no vs. yes), DM (no vs. yes), HDL (≥ 1.0 vs. < 1.0 mmol/L), eGFR (≥ 60 vs. < 60 ml/min/1.73 m^^2^) and antihypertensive medication (no vs. yes) (all *P-value* for interaction respectively were greater than 0.05; Additional file 4: Fig. 3).

## Discussion

In the present study, we showed an interesting finding that there was an inverse relationship of BMI levels with increased AS or baPWV, whereas WHR levels and central obesity were positively associated with increased AS or baPWV levels in Chinese rural adults with hypertension.

PWV was increased in hypertensive patients, and the degree of PWV increase was strongly linked to age and BP [11–13, 32, 40, 41]. In our study, nearly half (44.01%) of the hypertensive participants had increased AS (baPWV ≥ 18 m/s) and baPWV levels were also positively associated with age, SBP and DBP (Additional file 1: Table 1 and 3). From Table 1 follows that with increasing BMI, not only age decreased (6.78 years between the lowest and highest tertile of BMI levels), but also SBP significantly decreased (1.68 mmHg between the lowest and highest tertile of BMI levels). Even more important, DBP increased (4.52 mmHg between the lowest and highest tertile of BMI). On the contrary, concerning the comparison of the two WHR groups, there are differences in terms of SBP or DBP, and that age decreased only 0.82 year, probably not significantly. In consideration of that age and BP were closely associated with PWV levels [11–13, 32, 40, 41], stratified and interaction analyses were conducted to explore the effects of age, SBP, and DBP on the association between BMI or WHR levels and the prevalence of increased AS or baPWV (Additional file 1: Table 4–10 and Additional file 1: Fig. 3). There were no interactions in terms of age and BP on the relationship between BMI or WHR levels and the prevalence of increased AS or baPWV levels.

Hypertension is often accompanied with obesity [3]. In our study, we found that the prevalence of overweight, general obesity, central obesity and were 26.88%, 3.39% and 63.85%, respectively. In our analysis, only 5.12% of the 3, 224 patients with central obesity were general obesity according to BMI categories, but 96.49% of the 171 patients with general obesity were central obesity (Table 1 and Additional file 1: Table 2). In other words, the relative weight of general obesity in the group of elevated WHR is very small. Inversely, the relative weight of elevated WHR in the group of general obesity is very high. Considering the fact that the proportion of patients with general obesity is very small (3.39%) compared to the high proportion of subjects with central obesity (63.85%), which might explain why the correlation of BMI and WHR levels with the prevalence of increased AS based on baPWV were not coincident.

Similar to our results, Huang et al*.* [29] and Liu et al*.* [18] found that there was a negative relationship between the BMI levels and baPWV levels among male hypertension participants. However, previous studies also showed that the BMI levels were positively correlated with PWV levels in Grade I essential hypertension [15] or obese and non-obese hypertensive patients [17]. On the one hand, different origins of the study participants might contribute to this discrepancy. The Liu et al*.* [18] study was based on 699 male hypertensive patients who were hospitalization or had other complications. The Samir et al*.* [15] study was based on 114 civil servants with Grade I essential hypertension. The Huang et al*.* [29] study enrolled 101, 510 participants, a coal occupation group in labor-intensive enterprise, from 11 hospitals in the Kailuan community, which was most similar to our rural hypertensive patient populations. On the other hand, BMI is not able to differentiate fat-free muscle from fat mass. Furthermore, BMI cannot account for sex and racial differences in fat content, and it is not able to differentiate the distribution of visceral and subcutaneous fat [34]. The phenomenon of the obesity paradox may be related to genetics, cardiorespiratory fitness, beneficial adipose tissue and weaker sympathetic activation [34, 42, 43].

WHR, which is highly correlated with both increased visceral fat and low gluteal muscle mass [43–45], appears to be a more reliable prognosticator of CVDs than BMI [35, 36]. Previous studies found that a high WHR was strongly associated with increased AS based on PWV [19, 25, 27]. The Whitehall II study, a prospective study of 10 308 civil servants, showed that standardized effects of central adiposity on aortic PWV increase was obvious and previous adiposity was associated with aortic stiffening independent of change in adiposity, glycaemia, and lipid levels across PWV assessments [25]. We also demonstrated that WHR levels and central obesity were positively associated with increased AS or baPWV levels in Chinese rural adults with hypertension. Alexandre et al*.* [19] suggested that regional anthropometric indices including WHR were more closely correlated with PWV levels than BMI in hypertensive patients. Bouchi et al*.* [46], in a cross-sectional study with patients with diabetes and non-obese (normal BMI), noticed that increased visceral fat seems to be associated with increased AS based on baPWV. The lack of consistency between WHR and BMI may reflect that these measures identify different characteristics of obesity (central obesity in the case of WHR vs. subcutaneous/total fat in the case of BMI).

It is worth noting that eGFR was negatively associated with AS and baPWV in our study (Additional file 1: Table 1 and 3). Previous studies also showed that eGFR levels were reversely independently associated with cfPWV levels in patients with systemic lupus erythematosusa with a widely normally ranged eGFR levels (47). However, we cannot draw any causal relationship between eGFR levels and AS based on PWV in consideration of the cross-sectional analysis. Additionally, longitudinal studies indicated higher cfPWV levels were independently associated with steeper decline in eGFR levels and incident CKD in population more than 55 years old (48) and patients with type I diabetes (49). Higher cfPWV levels were also independently associated with the risk increased of progressive chronic kidney disease (CKD) in individuals with type II diabetes, and regression of PWV in the 3-year follow-up was associated with a lower risk of progressive CKD (50). With moderate progression of renal dysfunction and under well-controlled blood pressure during the 10-year follow-up period, peripheral AS based on baPWV but not central AS based on cfPWV was possibly one of the strongest predictors of CVD in patients with CKD stages III–V (51). These results suggest that AS based on PWV could be considered as a target for delaying decline in kidney function.

This study has several limitations. Firstly, this was a study of Chinese rural hypertensive patients and thus, the generalizability of the findings to other population remains to be determined. Secondly, the nutrional status such as the body shape might influence the measurement of baPWV causing information bias. Moreover, we cannot draw any causal relationship between the central obesity and AS based on baPWV considering the cross-sectional analysis. In the future, additional large-scale longitudinal studies are required to determine if weight loss programs reverse AS and reduce the risk of cardiovascular events.

## Conclusion

In conclusion, there was an inverse relationship between BMI levels and increased AS or baPWV, whereas WHR levels and central obesity were positively associated with increased AS or baPWV in Chinese rural adults with hypertension.

## Supplementary Information

**Additional file 1.** **Supplementary Table 1**. Association of covariates with the prevalence of increased arterial stiffness and baPWV levels. **Supplementary Table 2**. Relationship between BMI levels and WHR levels or central obesity. **Supplementary Table 3**. Clinical characteristics of participants grouped by baPWV quartiles. **Supplementary Table 4**. Relationship between BMI levels and the prevalence of increased arterial stiffness stratified by age. **Supplementary Table 5**. Relationship between BMI levels and the prevalence of increased arterial stiffness stratified by SBP. **Supplementary Table 6**. Relationship between BMI levels and the prevalence of increased arterial stiffness stratified by DBP. **Supplementary Table 7**. Relationship between WHR levels and the prevalence of increased arterial stiffness stratified by age. **Supplementary Table 8**. Relationship between WHR levels and the prevalence of increased arterial stiffness stratified by SBP. **Supplementary Table 9**. Relationship between WHR levels and the prevalence of increased arterial stiffness stratified by DBP. **Supplementary Table 10**. Effect size of BMI tertiles on baPWV levels in prespecified and exploratory subgroups.

**Additional file 2: Fig. ****1** The data flow chart of participants in our analysis. baPWV, brachial-ankle pulse wave velocity; BMI, body mass index; ABI, ankle brachial index; AF, atrial fibrillation; WHR, waist hip rate.

**Additional file 3: Fig. ****2** The correlation between BMI levels and WHR levels.

**Additional file 4: Fig. 3** Effect size of central obesity on baPWV levels in prespecified and exploratory subgroups. SBP, systolic blood pressure; DBP, diastolic blood pressure; DM, diabetes mellitus; HDL-C, high-density lipoprotein cholesterol; eGFR, estimated glomerular filtration rate*β*, effect size; CI, confidence interval. Each stratification adjusted for age, sex, SBP, DBP, HR, WHR, smoking and drinking status, homocysteine, ALT, eGFR, HDL-C, DM, CHD, history of stroke and antihypertensive medications except the subgroup variable.

## Data Availability

The datasets used and analysed during the current study available from the corresponding author on reasonable request.

## Abbreviations

CVDs: Cardiovascular diseases
AS: Arterial stiffness
cfPWV: Carotid-femoral pulse wave velocity
baPWV: Brachial-ankle pulse wave velocity
BMI: Body mass index
WHR: Waist hip rate
CHD: Coronary heart disease
SBP: Systolic blood pressure
DBP: Diastolic blood pressure
BP: Blood pressure
DM: Diabetes mellitus
FBG: Fasting blood glucose
TC: Total cholesterol
TG: Total triglyceride
HDL-C: High-density lipoprotein cholesterol
LDL-C: Low-density lipoprotein cholesterol
BUN: Blood urea nitrogen
eGFR: Estimated glomerular filtration rate
AST: Aspartate aminotransferase
ALT: Alanine aminotransferase
CKD-EPI: Chronic kidney disease epidemiology collaboration
CKD: Chronic kidney disease
SD: Standard deviation
OR: Odds ratio
CI: Confidence interval
