# Supplementary material for: Does body mass index or waist-hip ratio correlate with arterial stiffness based on brachial-ankle pulse wave velocity in Chinese rural adults with hypertension?
Source: BMC Cardiovasc Disord. 2021 Dec 1;21:573. doi: 10.1186/s12872-021-02390-y (PMC8638469; doi:10.1186/s12872-021-02390-y)
Supplement: Supplementary file 1 — Additional file 1. Supplementary Table 1. Association of covariates with the prevalence of increased arterial stiffness and baPWV levels. Supplementary Table 2. Relationship between BMI levels and WHR levels or central obesity. Supplementary Table 3. Clinical characteristics of participants grouped by baPWV quartiles. Supplementary Table 4. Relationship between BMI levels and the prevalence of increased arterial stiffness stratified by age. Supplementary Table 5. Relationship between BMI levels and the prevalence of increased arterial stiffness stratified by SBP. Supplementary Table 6. Relationship between BMI levels and the prevalence of increased arterial stiffness stratified by DBP. Supplementary Table 7. Relationship between WHR levels and the prevalence of increased arterial stiffness stratified by age. Supplementary Table 8. Relationship between WHR levels and the prevalence of increased arterial stiffness stratified by SBP. Supplementary Table 9. Relationship between WHR levels and the prevalence of increased arterial stiffness stratified by DBP. Supplementary Table 10. Effect size of BMI tertiles on baPWV levels in prespecified and exploratory subgroups. [file 12872_2021_2390_MOESM1_ESM.docx]

**Supplementary Table Legends**

| **Supplementary Table 1. Association of covariates with the prevalence of increased arterial stiffness and baPWV levels.** | | | | | |
| --- | --- | --- | --- | --- | --- |
| **Covariates** | **Increased AS** | |  | **baPWV** | |
|  | **OR (95%CI)** | ***P*-value** |  | **β (95%CI)** | ***P*-value** |
| Age (years) | 1.10 (1.09, 1.11) | <0.001 |  | 0.18 (0.17, 0.19) | <0.001 |
| Gender |  |  |  |  |  |
| Male | *Ref* |  |  | *Ref* |  |
| Female | 1.27 (1.13, 1.42) | <0.001 |  | 0.52 (0.31, 0.73) | <0.001 |
| SBP (mmHg) | 1.05 (1.05, 1.05) | <0.001 |  | 0.10 (0.09, 0.10) | <0.001 |
| DBP (mmHg) | 1.02 (1.01, 1.02) | <0.001 |  | 0.05 (0.04, 0.06) | <0.001 |
| HR (times/min) | 1.03 (1.02, 1.03) | <0.001 |  | 0.06 (0.06, 0.07) | <0.001 |
| BMI (kg/m^2) | 0.93 (0.91, 0.94) | <0.001 |  | -0.21 (-0.24, -0.18) | <0.001 |
| WHR | 4.44 (2.01, 9.79) | <0.001 |  | 1.72 (0.21, 3.22) | 0.026 |
| Smoking status, n(%) |  |  |  |  |  |
| Never | *Ref* |  |  | *Ref* |  |
| Former smoker | 1.04 (0.89, 1.20) | 0.627 |  | 0.12 (-0.17, 0.41) | 0.413 |
| Current smoker | 0.73 (0.64, 0.84) | <0.001 |  | -0.48 (-0.73, -0.23) | <0.001 |
| Drinking status, n(%) |  |  |  |  |  |
| Never | *Ref* |  |  | *Ref* |  |
| Former drinker | 0.99 (0.83, 1.18) | 0.893 |  | -0.12 (-0.46, 0.21) | 0.480 |
| Current drinker | 0.77 (0.67, 0.88) | <0.001 |  | -0.43 (-0.68, -0.17) | 0.001 |
| Homocysteine (μmol/L) | 1.01 (1.00, 1.01) | 0.003 |  | 0.02 (0.01, 0.03) | <0.001 |
| TC (mmol/L) | 1.12 (1.06, 1.17) | <0.001 |  | 0.20 (0.10, 0.29) | <0.001 |
| TG (mmol/L) | 1.01 (0.97, 1.06) | 0.600 |  | -0.00 (-0.09, 0.08) | 0.942 |
| HDL-C (mmol/L) | 1.23 (1.07, 1.41) | 0.004 |  | 0.65 (0.38, 0.92) | <0.001 |
| LDL-C (mmol/L) | 1.09 (1.02, 1.17) | 0.015 |  | 0.06 (-0.08, 0.19) | 0.394 |
| Serum uric acid (mmol/L) | 1.00 (1.00, 1.00) | 0.561 |  | -0.00 (-0.00, 0.00) | 0.577 |
| eGFR (ml/min/1.73m^^2^) | 0.98 (0.98, 0.98) | <0.001 |  | -0.04 (-0.05, -0.04) | <0.001 |
| AST (U/L) | 1.00 (1.00, 1.00) | 0.571 |  | 0.00 (-0.01, 0.01) | 0.641 |
| ALT (U/L) | 0.99 (0.99, 0.99) | <0.001 |  | -0.02 (-0.03, -0.01) | <0.001 |
| DM, n(%) |  |  |  |  |  |
| No | *Ref* |  |  | *Ref* |  |
| Yes | 1.59 (1.38, 1.83) | <0.001 |  | 0.80 (0.53, 1.08) | <0.001 |
| CHD, n(%) |  |  |  |  |  |
| No | *Ref* |  |  | *Ref* |  |
| Yes | 1.29 (1.04, 1.62) | 0.023 |  | 0.42 (-0.01, 0.85) | 0.053 |
| History of stroke, n(%) |  |  |  |  |  |
| No | *Ref* |  |  | *Ref* |  |
| Yes | 1.07 (0.87, 1.32) | 0.519 |  | 0.08 (-0.33, 0.49) | 0.704 |
| Antihypertensive medications, n(%) |  |  |  |  |  |
| No | *Ref* |  |  | *Ref* |  |
| Yes | 1.04 (0.93, 1.17) | 0.471 |  | 0.04 (-0.18, 0.26) | 0.744 |
| Lipid-lowering agents, n(%) |  |  |  |  |  |
| No | *Ref* |  |  | *Ref* |  |
| Yes | 0.94 (0.69, 1.27) | 0.674 |  | -0.58 (-1.17, 0.01) | 0.054 |
| Antiplatelet agents, n(%) |  |  |  |  |  |
| No | *Ref* |  |  | *Ref* |  |
| Yes | 1.17 (0.87, 1.58) | 0.288 |  | 0.06 (-0.51, 0.63) | 0.848 |
| Abbreviations: baPWV, brachial-ankle pulse wave velocity; BMI, body mass index; SBP, systolic blood pressure; DBP, diastolic blood pressure; HR, heart rate; WHR, waist hip rate; TC, total cholesterol; TG, total triglyceride; HDL-C, high-density lipoprotein cholesterol; LDL-C, low-density lipoprotein cholesterol; eGFR, estimated glomerular filtration rate; AST, aspartate aminotransferase; ALT, alanine aminotransferase; DM, diabetes mellitus; CHD, coronary heart disease. Values are regression coefficients from univariate regression models and reflect differences in the outcomes of interest per unit change of each covariate and for different categories of each covariate as compared to the reference group. | | | | | |

| **Supplementary Table 2. Relationship between BMI levels and WHR levels or central obesity.** | | | | | | | |
| --- | --- | --- | --- | --- | --- | --- | --- |
| **Variables** |  | **WHR** |  |  | **Central obesity** | | |
|  | **means ± SD** | ***β* (95%CI)** | ***P-value*** |  | **Event, n(%)** | **OR (95%CI)** | ***P-value*** |
| BMI (kg/m^^2^) |  |  |  |  |  |  |  |
| Per *SD* increase | 0.90 ± 0.07 | 0.04 (0.04, 0.04) | <0.001 |  | 3224 (63.85%) | 4.41 (4.02, 4.84) | <0.001 |
| BMI category (kg/m^^2^) |  |  |  |  |  |  |  |
| Control (<25) | 0.88 ± 0.06 | *Ref* |  |  | 1837 (52.17%) | *Ref* |  |
| Overweight (≥25, <30) | 0.94 ± 0.06 | 0.06 (0.06, 0.07) | <0.001 |  | 1222 (90.05%) | 8.30 (6.86, 10.03) | <0.001 |
| General obesity (≥30) | 0.97 ± 0.09 | 0.09 (0.08, 0.10) | <0.001 |  | 165 (96.49%) | 25.21 (11.14, 57.05) | <0.001 |
| *P-value* for trend |  | <0.001 | |  |  | <0.001 | |
| BMI tertiles (kg/m^^2^) |  |  |  |  |  |  |  |
| T1 [13.83, 21.68] | 0.85 ± 0.06 | *Ref* |  |  | *565 (33.57%)* | *Ref* |  |
| T2 [21.68, 24.69] | 0.90 ± 0.06 | 0.05 (0.05, 0.05) | <0.001 |  | 1143 (67.91%) | 4.19 (3.63, 4.84) | <0.001 |
| T3 [24.69, 46.43] | 0.94 ± 0.06 | 0.09 (0.09, 0.09) | <0.001 |  | 1516 (90.08%) | 17.96 (14.87, 21.70) | <0.001 |
| *P-value* for trend |  | <0.001 | |  |  | <0.001 | |
| Abbreviations: BMI, body mass index; WHR, waist hip rate; *Ref*, reference; *β*, effect size; OR, odds ratio; CI, confidence interval; *SD*, standard deviation. | | | | | | | |

| **Supplementary Table 3. Clinical characteristics of participants grouped by baPWV quartiles.** | | | | | | |
| --- | --- | --- | --- | --- | --- | --- |
| **Characteristics** | **Total** | **baPWV quartiles (m/s )** | | | | ***P-value*** |
|  |  | **Q1 [9.67, 15.36]** | **Q2 [15.37, 17.50]** | **Q3 [17.52, 20.06]** | **Q4 [20.07, 49.08]** |  |
| Number of subjects (n) | 5049 | 1262 | 1262 | 1261 | 1264 |  |
| Age (years) | 64.46 ± 9.45 | 58.74 ± 8.88 | 62.99 ± 8.53 | 66.09 ± 8.13 | 70.01 ± 8.44 | <0.001 |
| Male, n(%) | 2519 (49.89%) | 692 (54.83%) | 638 (50.55%) | 613 (48.61%) | 576 (45.57%) | <0.001 |
| SBP (mmHg) | 146.98 ± 17.56 | 137.07 ± 14.38 | 143.69 ± 14.97 | 150.33 ± 15.78 | 156.82 ± 18.34 | <0.001 |
| DBP (mmHg) | 88.70 ± 10.93 | 87.63 ± 9.96 | 87.88 ± 10.56 | 88.56 ± 11.06 | 90.75 ± 11.79 | <0.001 |
| HR (times/min) | 75.76 ± 14.37 | 72.46 ± 12.55 | 73.89 ± 12.28 | 76.42 ± 14.03 | 80.25 ± 16.92 | <0.001 |
| Height (cm) | 155.88 ± 8.17 | 158.27 ± 8.05 | 156.34 ± 7.90 | 155.23 ± 8.10 | 153.68 ± 7.95 | <0.001 |
| Weigh (Kg) | 56.87 ± 10.60 | 60.67 ± 10.57 | 57.33 ± 10.20 | 56.16 ± 10.25 | 53.32 ± 10.06 | <0.001 |
| BMI (kg/m^^2^) | 23.32 ± 3.49 | 24.16 ± 3.46 | 23.40 ± 3.45 | 23.24 ± 3.47 | 22.49 ± 3.38 | <0.001 |
| BMI group (kg/m^^2^) |  |  |  |  |  | <0.001 |
| Control (<25) | 3521 (69.74%) | 777 (61.57%) | 878 (69.57%) | 891 (70.66%) | 975 (77.14%) |  |
| Overweight (≥25, <30) | 1357 (26.88%) | 421 (33.36%) | 340 (26.94%) | 330 (26.17%) | 266 (21.04%) |  |
| General obesity (≥30) | 171 (3.39%) | 64 (5.07%) | 44 (3.49%) | 40 (3.17%) | 23 (1.82%) |  |
| Waistline (cm) | 82.32 ± 9.56 | 83.79 ± 9.45 | 82.23 ± 9.51 | 82.25 ± 9.62 | 81.01 ± 9.46 | <0.001 |
| Hipline (cm) | 91.49 ± 6.74 | 93.35 ± 6.59 | 91.79 ± 7.23 | 91.06 ± 6.39 | 89.76 ± 6.20 | <0.001 |
| WHR | 0.90 (0.85-0.94) | 0.90 (0.85-0.94) | 0.90 (0.85-0.94) | 0.90 (0.86-0.95) | 0.90 (0.85-0.95) | 0.021 |
| Central obesity, n(%) | 3224 (63.85%) | 792 (62.76%) | 771 (61.09%) | 820 (65.03%) | 841 (66.53%) | 0.023 |
| baPWV (m/s ) | 18.08 ± 3.87 | 13.98 ± 1.03 | 16.40 ± 0.61 | 18.69 ± 0.74 | 23.22 ± 3.40 | <0.001 |
| Increased AS, n(%) | 2222 (44.01%) | 0 (0.00%) | 0 (0.00%) | 958 (75.97%) | 1264 (100.00%) | <0.001 |
| Smoking status, n(%) |  |  |  |  |  | <0.001 |
| Never | 2691 (53.30%) | 666 (52.77%) | 636 (50.40%) | 685 (54.32%) | 704 (55.70%) |  |
| Former smoker | 932 (18.46%) | 211 (16.72%) | 223 (17.67%) | 242 (19.19%) | 256 (20.25%) |  |
| Current smoker | 1426 (28.24%) | 385 (30.51%) | 403 (31.93%) | 334 (26.49%) | 304 (24.05%) |  |
| Drinking status, n(%) |  |  |  |  |  | 0.009 |
| Never | 3194 (63.26%) | 764 (60.54%) | 791 (62.68%) | 809 (64.16%) | 830 (65.66%) |  |
| Former drinker | 609 (12.06%) | 147 (11.65%) | 143 (11.33%) | 171 (13.56%) | 148 (11.71%) |  |
| Current drinker | 1246 (24.68%) | 351 (27.81%) | 328 (25.99%) | 281 (22.28%) | 286 (22.63%) |  |
| Homocysteine (μmol/L) | 15.18 (12.58-19.64) | 14.32 (12.25-18.37) | 15.07 (12.47-19.26) | 15.23 (12.60-19.60) | 16.19 (13.14-21.00) | <0.001 |
| FBG (mmol/L) | 6.14 ± 1.63 | 5.84 ± 1.10 | 6.09 ± 1.71 | 6.25 ± 1.74 | 6.38 ± 1.82 | <0.001 |
| TC (mmol/L) | 5.12 ± 1.12 | 5.02 ± 1.09 | 5.09 ± 1.11 | 5.17 ± 1.12 | 5.22 ± 1.13 | <0.001 |
| TG (mmol/L) | 1.41 (1.00-2.08) | 1.40 (1.01-2.05) | 1.38 (0.98-2.04) | 1.43 (1.02-2.11) | 1.44 (1.02-2.08) | 0.326 |
| HDL-C (mmol/L) | 1.49 ± 0.40 | 1.46 ± 0.38 | 1.50 ± 0.41 | 1.49 ± 0.39 | 1.52 ± 0.41 | 0.006 |
| LDL-C (mmol/L) | 2.93 ± 0.80 | 2.89 ± 0.78 | 2.91 ± 0.79 | 2.96 ± 0.81 | 2.95 ± 0.81 | 0.078 |
| Serum uric acid (mmol/L) | 431.49 ± 121.34 | 434.56 ± 123.85 | 429.83 ± 124.54 | 432.39 ± 119.14 | 429.17 ± 117.78 | 0.508 |
| Serum creatinine (mmol/L) | 68.00 (56.00-83.00) | 67.00 (56.00-81.00) | 66.00 (56.00-81.00) | 69.00 (56.00-84.00) | 70.00 (57.00-88.00) | <0.001 |
| BUN (mmol/L) | 5.44 ± 1.83 | 5.33 ± 1.73 | 5.41 ± 1.69 | 5.49 ± 1.98 | 5.54 ± 1.92 | 0.032 |
| eGFR (ml/min/1.73m2) | 86.06 ± 19.61 | 91.89 ± 18.11 | 88.17 ± 18.57 | 84.21 ± 19.73 | 79.97 ± 19.97 | <0.001 |
| Total bilirubin (mmol/L) | 14.35 ± 6.34 | 14.53 ± 6.37 | 14.16 ± 6.00 | 14.38 ± 6.39 | 14.33 ± 6.58 | 0.561 |
| Direct bilirubin (mmol/L) | 5.36 ± 2.05 | 5.39 ± 1.99 | 5.32 ± 2.02 | 5.36 ± 2.06 | 5.37 ± 2.13 | 0.626 |
| AST (U/L) | 24.00 (20.00-30.00) | 24.00 (20.00-30.00) | 24.00 (20.00-30.00) | 24.00 (20.00-29.00) | 24.00 (20.00-30.00) | 0.760 |
| ALT (U/L) | 17.00 (12.00-24.00) | 18.00 (13.00-26.00) | 17.00 (13.00-25.00) | 16.00 (12.00-23.00) | 16.00 (12.00-23.00) | <0.001 |
| DM, n(%) | 935 (18.52%) | 163 (12.92%) | 210 (16.64%) | 272 (21.57%) | 290 (22.94%) | <0.001 |
| CHD, n(%) | 334 (6.62%) | 64 (5.07%) | 85 (6.74%) | 95 (7.53%) | 90 (7.12%) | 0.067 |
| History of stroke, n(%) | 375 (7.43%) | 88 (6.97%) | 95 (7.53%) | 100 (7.93%) | 92 (7.28%) | 0.825 |
| Dyslipidemia, n(%) | 1851 (36.66%) | 451 (35.74%) | 465 (36.85%) | 480 (38.07%) | 455 (36.00%) | 0.616 |
| Antihypertensive medications, n(%) | 3078 (60.96%) | 791 (62.68%) | 743 (58.87%) | 750 (59.48%) | 794 (62.82%) | 0.076 |
| Hypoglycemic agents, n(%) | 224 (4.44%) | 36 (2.85%) | 41 (3.25%) | 73 (5.79%) | 74 (5.85%) | <0.001 |
| Lipid-lowering agents, n(%) | 172 (3.41%) | 48 (3.80%) | 41 (3.25%) | 52 (4.12%) | 31 (2.45%) | 0.104 |
| Antiplatelet agents, n(%) | 184 (3.64%) | 42 (3.33%) | 47 (3.72%) | 52 (4.12%) | 43 (3.40%) | 0.701 |
| Abbreviations: BMI, body mass index; SBP, systolic blood pressure; DBP, diastolic blood pressure; HR, heart rate; WHR, waist hip rate; baPWV, brachial-ankle pulse wave velocity; AS, arterial stiffness; FBG, fasting blood glucose; TC, total cholesterol; TG, total triglyceride; HDL-C, high-density lipoprotein cholesterol; LDL-C, low-density lipoprotein cholesterol; BUN, blood urea nitrogen; eGFR, estimated glomerular filtration rate; AST, aspartate aminotransferase; ALT, alanine aminotransferase; DM, diabetes mellitus; CHD, coronary heart disease. | | | | | | |

| **Supplementary Table 4. Relationship between BMI levels and the prevalence of increased arterial stiffness stratified by age.** | | | | | | | | | |
| --- | --- | --- | --- | --- | --- | --- | --- | --- | --- |
| **Variables** | **Event, n(%)** | **Crude Model** | |  | **Model Ⅰ** | |  | **Model Ⅱ** | |
|  |  | **OR (95%CI)** | ***P-value*** |  | **OR (95%CI)** | ***P-value*** |  | **OR (95%CI)** | ***P-value*** |
| *Age tertiles (years)* |  |  |  |  |  |  |  |  |  |
| *Low [29.0, 60.0]* |  |  |  |  |  |  |  |  |  |
| BMI (kg/m^^2^) |  |  |  |  |  |  |  |  |  |
| Per *SD* increase | 335 (21.49%) | 0.88 (0.78, 1.00) | 0.056 |  | 0.77 (0.64, 0.92) | 0.004 |  | 0.72 (0.59, 0.86) | <0.001 |
| BMI category (kg/m^^2^) |  |  |  |  |  |  |  |  |  |
| Control (<25) | 195 (22.18%) | *Ref* |  |  | *Ref* |  |  | *Ref* |  |
| Overweight (≥25, <30) | 123 (20.92%) | 0.93 (0.72, 1.20) | 0.564 |  | 0.89 (0.64, 1.24) | 0.501 |  | 0.82 (0.59, 1.15) | 0.261 |
| General obesity (≥30) | 17 (18.48%) | 0.80 (0.46, 1.38) | 0.414 |  | 0.43 (0.21, 0.85) | 0.016 |  | 0.43 (0.21, 0.86) | 0.018 |
| *P-value* for trend |  | 0.364 | |  | 0.049 | |  | 0.027 | |
| BMI tertiles (kg/m^^2^) |  |  |  |  |  |  |  |  |  |
| T1 [14.72, 21.68] | 67 (25.09%) | *Ref* |  |  | *Ref* |  |  | *Ref* |  |
| T2 [21.68, 24.69] | 121 (21.88%) | 0.84 (0.59, 1.18) | 0.305 |  | 0.75 (0.49, 1.13) | 0.171 |  | 0.68 (0.44, 1.05) | 0.081 |
| T3 [24.69, 46.43] | 147 (19.89%) | 0.74 (0.53, 1.03) | 0.076 |  | 0.59 (0.38, 0.94) | 0.026 |  | 0.51 (0.32, 0.83) | 0.006 |
| *P-value* for trend |  | 0.076 | |  | 0.025 | |  | 0.006 | |
| *Middle [61.0, 68.0]* |  |  |  |  |  |  |  |  |  |
| BMI (kg/m^^2^) |  |  |  |  |  |  |  |  |  |
| Per *SD* increase | 773 (43.23%) | 1.01 (0.91, 1.11) | 0.914 |  | 0.86 (0.74, 0.99) | 0.036 |  | 0.78 (0.67, 0.91) | 0.001 |
| BMI category (kg/m^^2^) |  |  |  |  |  |  |  |  |  |
| Control (<25) | 558 (43.49%) | *Ref* |  |  | *Ref* |  |  | *Ref* |  |
| Overweight (≥25, <30) | 193 (42.79%) | 0.97 (0.78, 1.21) | 0.797 |  | 0.81 (0.62, 1.07) | 0.133 |  | 0.74 (0.56, 0.97) | 0.032 |
| General obesity (≥30) | 22 (40.74%) | 0.89 (0.51, 1.55) | 0.690 |  | 0.70 (0.36, 1.35) | 0.286 |  | 0.59 (0.30, 1.17) | 0.130 |
| *P-value* for trend |  | 0.669 | |  | 0.093 | |  | 0.017 | |
| BMI tertiles (kg/m^^2^) |  |  |  |  |  |  |  |  |  |
| T1 [14.40, 21.68] | 267 (43.56%) | *Ref* |  |  | *Ref* |  |  | *Ref* |  |
| T2 [21.69, 24.68] | 260 (42.41%) | 0.95 (0.76, 1.20) | 0.686 |  | 0.84 (0.63, 1.11) | 0.215 |  | 0.75 (0.56, 0.99) | 0.044 |
| T3 [24.70, 40.84] | 246 (43.77%) | 1.01 (0.80, 1.27) | 0.941 |  | 0.78 (0.56, 1.07) | 0.124 |  | 0.64 (0.46, 0.90) | 0.010 |
| *P-value* for trend |  | 0.952 | |  | 0.126 | |  | 0.010 | |
| *High [69.0, 93.0]* |  |  |  |  |  |  |  |  |  |
| BMI (kg/m^^2^) |  |  |  |  |  |  |  |  |  |
| Per *SD* increase | 1114 (65.45%) | 0.90 (0.81, 1.00) | 0.058 |  | 0.78 (0.67, 0.91) | 0.001 |  | 0.69 (0.59, 0.82) | <0.001 |
| *P-value* for interaction |  | 0.190 | |  | 0.561 | |  | 0.543 | |
| BMI category (kg/m^^2^) |  |  |  |  |  |  |  |  |  |
| Control (<25) | 900 (66.23%) | *Ref* |  |  | *Ref* |  |  | *Ref* |  |
| Overweight (≥25, <30) | 198 (62.26% | 0.84 (0.65, 1.08) | 0.181 |  | 0.69 (0.50, 0.94) | 0.019 |  | 0.60 (0.44, 0.83) | 0.002 |
| General obesity (≥30) | 16 (64.00%) | 0.91 (0.40, 2.07) | 0.816 |  | 0.60 (0.24, 1.50) | 0.274 |  | 0.53 (0.21, 1.35) | 0.183 |
| *P-value* for trend |  | 0.218 | |  | 0.014 | |  | 0.002 | |
| *P-value* for interaction |  | 0.932 | |  | 0.402 | |  | 0.436 | |
| BMI tertiles (kg/m^^2^) |  |  |  |  |  |  |  |  |  |
| T1 [13.83, 21.68] | 543 (67.62%) | *Ref* |  |  | *Ref* |  |  | *Ref* |  |
| T2 [21.69, 24.69] | 334 (64.60%) | 0.87 (0.69, 1.10) | 0.257 |  | 0.83 (0.62, 1.11) | 0.209 |  | 0.71 (0.53, 0.96) | 0.024 |
| T3 [24.70, 38.76] | 237 (62.04%) | 0.78 (0.61, 1.01) | 0.059 |  | 0.62 (0.44, 0.87) | 0.006 |  | 0.48 (0.34, 0.69) | <0.001 |
| *P-value* for trend |  | 0.052 | |  | 0.007 | |  | <0.001 | |
| *P-value* for interaction |  | 0.521 | |  | 0.783 | |  | 0.770 | |
| Abbreviations: BMI, body mass index; *Ref*, reference; OR, odds ratio; CI, confidence interval; *SD*, standard deviation. ModelⅠadjusted for age, sex, SBP, DBP, HR and WHR. ModelⅡadjusted for age, sex, SBP, DBP, HR, WHR, smoking status, TG, LDL-C, eGFR, DM, CHD, history of stroke and antihypertensive medications. | | | | | | | | | |

| **Supplementary Table 5. Relationship between BMI levels and the prevalence of increased arterial stiffness stratified by SBP.** | | | | | | | | | |
| --- | --- | --- | --- | --- | --- | --- | --- | --- | --- |
| **Variables** | **Event, n(%)** | **Crude Model** | |  | **Model Ⅰ** | |  | **Model Ⅱ** | |
|  |  | **OR (95%CI)** | ***P-value*** |  | **OR (95%CI)** | ***P-value*** |  | **OR (95%CI)** | ***P-value*** |
| *SBP tertiles (mmHg)* |  |  |  |  |  |  |  |  |  |
| *Low [83.33, 139.00]* |  |  |  |  |  |  |  |  |  |
| BMI (kg/m^^2^) |  |  |  |  |  |  |  |  |  |
| Per *SD* increase | 420 (25.01%) | 0.72 (0.64, 0.80) | <0.001 |  | 0.62 (0.53, 0.73) | <0.001 |  | 0.59 (0.50, 0.71) | <0.001 |
| BMI category (kg/m^^2^) |  |  |  |  |  |  |  |  |  |
| Control (<25) | 317 (27.95%) | *Ref* |  |  | *Ref* |  |  | *Ref* |  |
| Overweight (≥25, <30) | 98 (20.08%) | 0.65 (0.50, 0.84) | <0.001 |  | 0.59 (0.43, 0.79) | <0.001 |  | 0.55 (0.40, 0.75) | <0.001 |
| General obesity (≥30) | 5 (8.77%) | 0.25 (0.10, 0.63) | 0.003 |  | 0.20 (0.07, 0.54) | 0.002 |  | 0.21 (0.08, 0.57) | 0.002 |
| *P-value* for trend |  | <0.001 | |  | 0.015 | |  | 0.003 | |
| BMI tertiles (kg/m^^2^) |  |  |  |  |  |  |  |  |  |
| T1 [13.94, 21.68] | 165 (31.61%) | *Ref* |  |  | *Ref* |  |  | *Ref* |  |
| T2 [21.68, 24.69] | 142 (25.59%) | 0.74 (0.57, 0.97) | 0.029 |  | 0.72 (0.52, 1.00) | 0.047 |  | 0.70 (0.51, 0.98) | 0.037 |
| T3 [24.69, 40.84] | 113 (18.77%) | 0.50 (0.38, 0.66) | <0.001 |  | 0.43 (0.30, 0.62) | <0.001 |  | 0.39 (0.27, 0.57) | <0.001 |
| *P-value* for trend |  | <0.001 | |  | 0.034 | |  | 0.003 | |
| *Middle [139.33, 153.00]* |  |  |  |  |  |  |  |  |  |
| BMI (kg/m^^2^) |  |  |  |  |  |  |  |  |  |
| Per *SD* increase | 682 (40.96%) | 0.78 (0.71, 0.87) | <0.001 |  | 0.68 (0.59, 0.78) | <0.001 |  | 0.65 (0.56, 0.75) | <0.001 |
| BMI category (kg/m^^2^) |  |  |  |  |  |  |  |  |  |
| Control (<25) | 502 (43.54%) | *Ref* |  |  | *Ref* |  |  | *Ref* |  |
| Overweight (≥25, <30) | 157 (34.73%) | 0.69 (0.55, 0.87) | 0.001 |  | 0.62 (0.48, 0.81) | <0.001 |  | 0.58 (0.44, 0.77) | <0.001 |
| General obesity (≥30) | 23 (38.33%) | 0.81 (0.47, 1.37) | 0.428 |  | 0.62 (0.35, 1.11) | 0.109 |  | 0.64 (0.35, 1.16) | 0.138 |
| *P-value* for trend |  | 0.005 | |  | 0.243 | |  | 0.095 | |
| BMI tertiles (kg/m^^2^) |  |  |  |  |  |  |  |  |  |
| T1 [14.09, 21.67] | 269 (49.09%) | *Ref* |  |  | *Ref* |  |  | *Ref* |  |
| T2 [21.69, 24.68] | 218 (38.86% | 0.66 (0.52, 0.84) | <0.001 |  | 0.60 (0.46, 0.79) | <0.001 |  | 0.56 (0.42, 0.75) | <0.001 |
| T3 [24.69, 42.01] | 195 (35.07%) | 0.56 (0.44, 0.71) | <0.001 |  | 0.44 (0.32, 0.60) | <0.001 |  | 0.38 (0.27, 0.53) | <0.001 |
| *P-value* for trend |  | <0.001 | |  | 0.101 | |  | 0.007 | |
| *High [153.33, 255.00]* |  |  |  |  |  |  |  |  |  |
| BMI (kg/m^^2^) |  |  |  |  |  |  |  |  |  |
| Per *SD* increase | 1120 (65.69%) | 0.80 (0.72, 0.88) | <0.001 |  | 0.59 (0.52, 0.69) | <0.001 |  | 0.59 (0.50, 0.68) | <0.001 |
| *P-value* for interaction |  | 0.341 | |  | 0.151 | |  | 0.171 | |
| BMI category (kg/m^^2^) |  |  |  |  |  |  |  |  |  |
| Control (<25) | 834 (67.59%) | *Ref* |  |  | *Ref* |  |  | *Ref* |  |
| Overweight (≥25, <30) | 259 (62.11%) | 0.79 (0.62, 0.99) | 0.041 |  | 0.57 (0.44, 0.76) | <0.001 |  | 0.57 (0.43, 0.75) | <0.001 |
| General obesity (≥30) | 27 (50.00%) | 0.48 (0.28, 0.83) | 0.008 |  | 0.26 (0.14, 0.48) | <0.001 |  | 0.27 (0.14, 0.51) | <0.001 |
| *P-value* for trend |  | 0.002 | |  | 0.012 | |  | 0.004 | |
| *P-value* for interaction |  | 0.143 | |  | 0.299 | |  | 0.270 | |
| BMI tertiles (kg/m^^2^) |  |  |  |  |  |  |  |  |  |
| T1 [13.83, 21.68] | 443 (72.27%) | *Ref* |  |  | *Ref* |  |  | *Ref* |  |
| T2 [21.68, 24.69] | 355 (62.61%) | 0.64 (0.50, 0.82) | <0.001 |  | 0.48 (0.36, 0.64) | <0.001 |  | 0.48 (0.35, 0.64) | <0.001 |
| T3 [24.70, 46.43] | 322 (61.33%) | 0.61 (0.47, 0.78) | <0.001 |  | 0.33 (0.24, 0.46) | <0.001 |  | 0.32 (0.23, 0.45) | <0.001 |
| *P-value* for trend |  | <0.001 | |  | 0.003 | |  | <0.001 | |
| *P-value* for interaction |  | 0.495 | |  | 0.598 | |  | 0.566 | |
| Abbreviations: SBP, systolic blood pressure; BMI, body mass index; *Ref*, reference; OR, odds ratio; CI, confidence interval; *SD*, standard deviation. ModelⅠadjusted for age, sex, SBP, DBP, HR and WHR. ModelⅡadjusted for age, sex, SBP, DBP, HR, WHR, smoking status, TG, LDL-C, eGFR, DM, CHD, history of stroke and antihypertensive medications. | | | | | | | | | |

| **Supplementary Table 6. Relationship between BMI levels and the prevalence of increased arterial stiffness stratified by DBP.** | | | | | | | | | |  |
| --- | --- | --- | --- | --- | --- | --- | --- | --- | --- | --- |
| **Variables** | **Event, n(%)** | **Crude Model** | |  | **Model Ⅰ** | |  | **Model Ⅱ** | | |
|  |  | **OR (95%CI)** | ***P-value*** |  | **OR (95%CI)** | ***P-value*** |  | **OR (95%CI)** | ***P-value*** | |
| *DBP tertiles (mmHg)* |  |  |  |  |  |  |  |  |  | |
| *Low [41.67, 83.67]* |  |  |  |  |  |  |  |  |  | |
| BMI (kg/m^^2^) |  |  |  |  |  |  |  |  |  | |
| Per *SD* increase | 689 (41.94%) | 0.80 (0.72, 0.88) | <0.001 |  | 0.70 (0.60, 0.80) | <0.001 |  | 0.65 (0.56, 0.76) | <0.001 | |
| BMI category (kg/m^^2^) |  |  |  |  |  |  |  |  |  | |
| Control (<25) | 554 (43.86%) | *Ref* |  |  | *Ref* |  |  | *Ref* |  | |
| Overweight (≥25, <30) | 124 (35.53%) | 0.71 (0.55, 0.90) | 0.005 |  | 0.60 (0.45, 0.81) | <0.001 |  | 0.55 (0.40, 0.75) | <0.001 | |
| General obesity (≥30) | 11 (35.48%) | 0.70 (0.33, 1.48) | 0.355 |  | 0.51 (0.23, 1.17) | 0.111 |  | 0.51 (0.22, 1.17) | 0.110 | |
| *P-value* for trend |  | <0.001 | |  | 0.048 | |  | 0.011 | | |
| BMI tertiles (kg/m^^2^) |  |  |  |  |  |  |  |  |  | |
| T1 [13.94, 21.68] | 338 (47.94%) | *Ref* |  |  | *Ref* |  |  | *Ref* |  | |
| T2 [21.69, 24.68] | 205 (39.58%) | 0.71 (0.57, 0.89) | 0.004 |  | 0.67 (0.51, 0.88) | 0.004 |  | 0.60 (0.45, 0.80) | <0.001 | |
| T3 [24.70, 35.61] | 146 (34.76%) | 0.58 (0.45, 0.74) | <0.001 |  | 0.45 (0.32, 0.62) | <0.001 |  | 0.38 (0.27, 0.53) | <0.001 | |
| *P-value* for trend |  | <0.001 | |  | 0.008 | |  | <0.001 | | |
| *Middle [84.00, 92.67]* |  |  |  |  |  |  |  |  |  | |
| BMI (kg/m^^2^) |  |  |  |  |  |  |  |  |  | |
| Per *SD* increase | 708 (42.17%) | 0.78 (0.71, 0.87) | <0.001 |  | 0.67 (0.57, 0.78) | <0.001 |  | 0.62 (0.53, 0.73) | <0.001 | |
| BMI category (kg/m^^2^) |  |  |  |  |  |  |  |  |  | |
| Control (<25) | 521 (44.84%) | *Ref* |  |  | *Ref* |  |  | *Ref* |  | |
| Overweight (≥25, <30) | 171 (37.09%) | 0.73 (0.58, 0.91) | 0.005 |  | 0.65 (0.49, 0.86) | 0.003 |  | 0.60 (0.44, 0.80) | <0.001 | |
| General obesity (≥30) | 16 (28.57%) | 0.49 (0.27, 0.89) | 0.019 |  | 0.44 (0.22, 0.90) | 0.025 |  | 0.39 (0.18, 0.83) | 0.015 | |
| *P-value* for trend |  | 0.005 | |  | 0.121 | |  | 0.033 | | |
| BMI tertiles (kg/m^^2^) |  |  |  |  |  |  |  |  |  | |
| T1 [14.32, 21.67] | 257 (49.33%) | *Ref* |  |  | *Ref* |  |  | *Ref* |  | |
| T2 [21.69, 24.69] | 241 (41.27%) | 0.72 (0.57, 0.92) | 0.007 |  | 0.65 (0.48, 0.88) | 0.005 |  | 0.63 (0.46, 0.85) | 0.003 | |
| T3 [24.69, 40.84] | 210 (36.59%) | 0.59 (0.47, 0.75) | <0.001 |  | 0.46 (0.33, 0.65) | <0.001 |  | 0.40 (0.28, 0.57) | <0.001 | |
| *P-value* for trend |  | <0.001 | |  | 0.157 | |  | 0.020 | | |
| *High [93.00, 129.67]* |  |  |  |  |  |  |  |  |  | |
| BMI (kg/m^^2^) |  |  |  |  |  |  |  |  |  | |
| Per *SD* increase | 825 (47.77%) | 0.68 (0.62, 0.76) | <0.001 |  | 0.54 (0.46, 0.62) | <0.001 |  | 0.54 (0.46, 0.64) | <0.001 | |
| *P-value* for interaction |  | 0.080 | |  | 0.345 | |  | 0.234 | | |
| BMI category (kg/m^^2^) |  |  |  |  |  |  |  |  |  | |
| Control (<25) | 578 (52.74%) | *Ref* |  |  | *Ref* |  |  | *Ref* |  | |
| Overweight (≥25, <30) | 219 (40.04%) | 0.60 (0.49, 0.74) | <0.001 |  | 0.53 (0.40, 0.68) | <0.001 |  | 0.52 (0.40, 0.69) | <0.001 | |
| General obesity (≥30) | 28 (33.33%) | 0.45 (0.28, 0.72) | <0.001 |  | 0.24 (0.13, 0.42) | <0.001 |  | 0.27 (0.15, 0.49) | <0.001 | |
| *P-value* for trend |  | 0.002 | |  | 0.005 | |  | <0.001 | | |
| *P-value* for interaction |  | 0.619 | |  | 0.509 | |  | 0.537 | | |
| BMI tertiles (kg/m^^2^) |  |  |  |  |  |  |  |  |  | |
| T1 [13.83, 21.68] | 282 (61.71%) | *Ref* |  |  | *Ref* |  |  | *Ref* |  | |
| T2 [21.68, 24.69] | 269 (46.30%) | 0.54 (0.42, 0.69) | <0.001 |  | 0.45 (0.33, 0.61) | <0.001 |  | 0.48 (0.35, 0.67) | <0.001 | |
| T3 [24.70, 46.43] | 274 (39.77%) | 0.41 (0.32, 0.52) | <0.001 |  | 0.28 (0.20, 0.39) | <0.001 |  | 0.29 (0.20, 0.42) | <0.001 | |
| *P-value* for trend |  | <0.001 | |  | 0.009 | |  | 0.001 | | |
| *P-value* for interaction |  | 0.189 | |  | 0.432 | |  | 0.352 | | |
| Abbreviations: DBP, diastolic blood pressure; BMI, body mass index; *Ref*, reference; OR, odds ratio; CI, confidence interval; *SD*, standard deviation. ModelⅠadjusted for age, sex, SBP, DBP, HR and WHR. ModelⅡadjusted for age, sex, SBP, DBP, HR, WHR, smoking status, TG, LDL-C, eGFR, DM, CHD, history of stroke and antihypertensive medications. | | | | | | | | | | |

| **Supplementary Table 7. Relationship between WHR levels and the prevalence of increased arterial stiffness stratified by age.** | | | | | | | | | |
| --- | --- | --- | --- | --- | --- | --- | --- | --- | --- |
| **Variables** | **Event, n(%)** | **Crude Model** | |  | **Model Ⅰ** | |  | **Model Ⅱ** | |
|  |  | **OR (95%CI)** | ***P-value*** |  | **OR (95%CI)** | ***P-value*** |  | **OR (95%CI)** | ***P-value*** |
| *Age tertiles (years)* |  |  |  |  |  |  |  |  |  |
| *Low [29.0, 60.0]* |  |  |  |  |  |  |  |  |  |
| WHR |  |  |  |  |  |  |  |  |  |
| Per *SD* increase | 335 (21.49%) | 1.17 (1.02, 1.33) | 0.020 |  | 1.42 (1.18, 1.71) | <0.001 |  | 1.38 (1.14, 1.67) | <0.001 |
| Central obesity |  |  |  |  |  |  |  |  |  |
| No | 95 (18.52%) | *Ref* |  |  | *Ref* |  |  | *Ref* |  |
| Yes | 240 (22.94%) | 1.31 (1.00, 1.71) | 0.046 |  | 1.83 (1.28, 2.60) | <0.001 |  | 1.74 (1.20, 2.50) | 0.003 |
| WHR tertiles |  |  |  |  |  |  |  |  |  |
| T1 [0.58, 0.87] | 98 (19.92%) | *Ref* |  |  | *Ref* |  |  | *Ref* |  |
| T2 [0.87, 0.93] | 115 (20.72%) | 1.05 (0.78, 1.42) | 0.748 |  | 1.39 (0.96, 2.02) | 0.085 |  | 1.26 (0.86, 1.86) | 0.242 |
| T3 [0.93, 1.41] | 122 (23.83%) | 1.26 (0.93, 1.70) | 0.135 |  | 2.06 (1.35, 3.15) | <0.001 |  | 1.88 (1.22, 2.91) | 0.005 |
| *P-value* for trend |  | 0.134 | |  | <0.001 | |  | 0.004 | |
| *Middle [61.0, 68.0]* |  |  |  |  |  |  |  |  |  |
| WHR |  |  |  |  |  |  |  |  |  |
| Per *SD* increase | 773 (43.23%) | 1.17 (1.06, 1.29) | 0.001 |  | 1.25 (1.09, 1.44) | 0.002 |  | 1.21 (1.06, 1.40) | 0.006 |
| Central obesity |  |  |  |  |  |  |  |  |  |
| No | 250 (37.88%) | *Ref* |  |  | *Ref* |  |  | *Ref* |  |
| Yes | 523 (46.37%) | 1.42 (1.17, 1.72) | <0.001 |  | 1.41 (1.08, 1.85) | 0.013 |  | 1.34 (1.02, 1.77) | 0.037 |
| WHR tertiles |  |  |  |  |  |  |  |  |  |
| T1 [0.59, 0.87] | 237 (39.83%) | *Ref* |  |  | *Ref* |  |  | *Ref* |  |
| T2 [0.87, 0.93] | 248 (41.06%) | 1.05 (0.84, 1.33) | 0.665 |  | 1.15 (0.87, 1.53) | 0.328 |  | 1.14 (0.85, 1.51) | 0.386 |
| T3 [0.93, 1.68] | 288 (48.90%) | 1.45 (1.15, 1.82) | 0.002 |  | 1.65 (1.20, 2.29) | 0.002 |  | 1.55 (1.11, 2.16) | 0.009 |
| *P-value* for trend |  | 0.002 | |  | 0.002 | |  | 0.009 | |
| *High [69.0, 93.0]* |  |  |  |  |  |  |  |  |  |
| WHR |  |  |  |  |  |  |  |  |  |
| Per *SD* increase | 588 (34.55%) | 1.08 (0.98, 1.19) | 0.106 |  | 1.22 (1.07, 1.40) | 0.004 |  | 1.22 (1.06, 1.40) | 0.005 |
| *P-value* for interaction |  | 0.450 | |  | 0.338 | |  | 0.396 | |
| Central obesity |  |  |  |  |  |  |  |  |  |
| No | 398 (61.04%) | *Ref* |  |  | *Ref* |  |  | *Ref* |  |
| Yes | 716 (68.19%) | 1.37 (1.12, 1.68) | 0.003 |  | 1.68 (1.27, 2.23) | <0.001 |  | 1.63 (1.22, 2.17) | <0.001 |
| *P-value* for interaction |  | 0.895 | |  | 0.871 | |  | 0.840 | |
| WHR tertiles |  |  |  |  |  |  |  |  |  |
| T1 [0.53, 0.87] | 362 (60.74%) | *Ref* |  |  | *Ref* |  |  | *Ref* |  |
| T2 [0.87, 0.93] | 353 (68.15%) | 1.38 (1.08, 1.77) | 0.010 |  | 1.81 (1.34, 2.46) | <0.001 |  | 1.81 (1.33, 2.47) | <0.001 |
| T3 [0.93, 1.65] | 399 (67.86%) | 1.36 (1.07, 1.73) | 0.011 |  | 2.13 (1.53, 2.97) | <0.001 |  | 2.11 (1.50, 2.96) | <0.001 |
| *P-value* for trend |  | <0.001 | |  | <0.001 | |  | <0.001 | |
| *P-value* for interaction |  | 0.318 | |  | 0.438 | |  | 0.478 | |
| Abbreviations: WHR, waist hip rate; *Ref*, reference; OR, odds ratio; CI, confidence interval; *SD*, standard deviation. ModelⅠadjusted for age, sex, SBP, DBP, HR and BMI. ModelⅡadjusted for age, sex, SBP, DBP, HR, BMI, smoking status, ALT, HDL-C, eGFR, DM, CHD, history of stroke and antihypertensive medications. | | | | | | | | | |

| **Supplementary Table 8. Relationship between WHR levels and the prevalence of increased arterial stiffness stratified by SBP.** | | | | | | | | | |
| --- | --- | --- | --- | --- | --- | --- | --- | --- | --- |
| **Variables** | **Event, n(%)** | **Crude Model** | |  | **Model Ⅰ** | |  | **Model Ⅱ** | |
|  |  | **OR (95%CI)** | ***P-value*** |  | **OR (95%CI)** | ***P-value*** |  | **OR (95%CI)** | ***P-value*** |
| *SBP tertiles (mmHg)* |  |  |  |  |  |  |  |  |  |
| *Low [83.33, 139.00]* |  |  |  |  |  |  |  |  |  |
| WHR |  |  |  |  |  |  |  |  |  |
| Per *SD* increase | 420 (25.01%) | 1.06 (0.95, 1.18) | 0.283 |  | 1.21 (1.04, 1.41) | 0.012 |  | 1.18 (1.02, 1.38) | 0.030 |
| Central obesity |  |  |  |  |  |  |  |  |  |
| No | 143 (23.83%) | *Ref* |  |  | *Ref* |  |  | *Ref* |  |
| Yes | 277 (25.67%) | 1.10 (0.88, 1.39) | 0.405 |  | 1.60 (1.14, 2.22) | 0.006 |  | 1.47 (1.04, 2.07) | 0.028 |
| WHR tertiles |  |  |  |  |  |  |  |  |  |
| T1 [0.53, 0.87] | 133 (24.49%) | *Ref* |  |  | *Ref* |  |  | *Ref* |  |
| T2 [0.87, 0.93] | 128 (23.40%) | 0.94 (0.71, 1.24) | 0.672 |  | 1.40 (0.98, 2.00) | 0.0613 |  | 1.31 (0.91, 1.88) | 0.150 |
| T3 [0.93, 1.68] | 159 (26.99%) | 1.14 (0.87, 1.49) | 0.337 |  | 1.60 (1.09, 2.36) | 0.018 |  | 1.49 (1.00, 2.22) | 0.049 |
| *P-value* for trend |  | 0.333 | |  | 0.019 | |  | 0.052 | |
| *Middle [139.33, 153.00]* |  |  |  |  |  |  |  |  |  |
| WHR |  |  |  |  |  |  |  |  |  |
| Per *SD* increase | 682 (40.96%) | 1.14 (1.03, 1.26) | 0.012 |  | 1.23 (1.06, 1.42) | 0.005 |  | 1.17 (1.01, 1.35) | 0.042 |
| Central obesity |  |  |  |  |  |  |  |  |  |
| No | 217 (36.35%) | *Ref* |  |  | *Ref* |  |  | *Ref* |  |
| Yes | 465 (43.54%) | 1.35 (1.10, 1.66) | 0.004 |  | 1.69 (1.28, 2.24) | <0.001 |  | 1.55 (1.16, 2.06) | 0.003 |
| WHR tertiles |  |  |  |  |  |  |  |  |  |
| T1 [0.59, 0.87] | 199 (36.25%) | *Ref* |  |  | *Ref* |  |  | *Ref* |  |
| T2 [0.87, 0.93] | 220 (39.15%) | 1.13 (0.89, 1.44) | 0.319 |  | 1.45 (1.08, 1.95) | 0.013 |  | 1.39 (1.03, 1.88) | 0.034 |
| T3 [0.93, 1.65] | 263 (47.47%) | 1.59 (1.25, 2.02) | <0.001 |  | 2.15 (1.54, 3.00) | <0.001 |  | 1.91 (1.35, 2.69) | <0.001 |
| *P-value* for trend |  | <0.001 | |  | <0.001 | |  | <0.001 | |
| *High [153.33, 255.00]* |  |  |  |  |  |  |  |  |  |
| WHR |  |  |  |  |  |  |  |  |  |
| Per *SD* increase | 1120 (65.69%) | 1.21 (1.09, 1.35) | <0.001 |  | 1.43 (1.23, 1.66) | <0.001 |  | 1.42 (1.22, 1.66) | <0.001 |
| *P-value* for interaction |  | 0.210 | |  | 0.120 | |  | 0.109 | |
| Central obesity |  |  |  |  |  |  |  |  |  |
| No | 383 (60.99%) | *Ref* |  |  | *Ref* |  |  | *Ref* |  |
| Yes | 737 (68.43%) | 1.39 (1.13, 1.70) | 0.002 |  | 1.59 (1.20, 2.09) | 0.001 |  | 1.53 (1.15, 2.03) | 0.004 |
| *P-value* for interaction |  | 0.304 | |  | 0.420 | |  | 0.349 | |
| WHR tertiles |  |  |  |  |  |  |  |  |  |
| T1 [0.60, 0.87] | 365 (61.76%) | *Ref* |  |  | *Ref* |  |  | *Ref* |  |
| T2 [0.87, 0.93] | 368 (64.79%) | 1.14 (0.90, 1.45) | 0.285 |  | 1.40 (1.05, 1.88) | 0.023 |  | 1.36 (1.01, 1.84) | 0.042 |
| T3 [0.93, 1.35] | 387 (70.88%) | 1.51 (1.18, 1.93) | 0.001 |  | 2.06 (1.46, 2.90) | <0.001 |  | 2.01 (1.42, 2.85) | <0.001 |
| *P-value* for trend |  | <0.001 | |  | <0.001 | |  | <0.001 |  |
| *P-value* for interaction |  | 0.442 | |  | 0.409 | |  | 0.415 | |
| Abbreviations: SBP, systolic blood pressure; WHR, waist hip rate; *Ref*, reference; OR, odds ratio; CI, confidence interval; *SD*, standard deviation. ModelⅠadjusted for age, sex, SBP, DBP, HR and BMI. ModelⅡadjusted forage, sex, SBP, DBP, HR, BMI, smoking status, ALT, HDL-C, eGFR, DM, CHD, history of stroke and antihypertensive medications. | | | | | | | | | |

| **Supplementary Table 9. Relationship between WHR levels and the prevalence of increased arterial stiffness stratified by DBP.** | | | | | | | | | |
| --- | --- | --- | --- | --- | --- | --- | --- | --- | --- |
| **Variables** | **Event, n(%)** | **Crude Model** | |  | **Model Ⅰ** | |  | **Model Ⅱ** | |
|  |  | **OR (95%CI)** | ***P-value*** |  | **OR (95%CI)** | ***P-value*** |  | **OR (95%CI)** | ***P-value*** |
| *DBP tertiles (mmHg)* |  |  |  |  |  |  |  |  |  |
| *Low [41.67, 83.67]* |  |  |  |  |  |  |  |  |  |
| WHR |  |  |  |  |  |  |  |  |  |
| Per *SD* increase | 689 (41.94%) | 1.03 (0.94, 1.13) | 0.535 |  | 1.11 (0.98, 1.27) | 0.111 |  | 1.10 (0.96, 1.26) | 0.170 |
| Central obesity |  |  |  |  |  |  |  |  |  |
| No | 246 (39.30%) | *Ref* |  |  | *Ref* |  |  | *Ref* |  |
| Yes | 443 (43.56%) | 1.19 (0.97, 1.46) | 0.089 |  | 1.50 (1.12, 1.99) | 0.006 |  | 1.43 (1.07, 1.92) | 0.016 |
| WHR tertiles |  |  |  |  |  |  |  |  |  |
| T1 [0.53, 0.87] | 240 (39.67%) | *Ref* |  |  | *Ref* |  |  | *Ref* |  |
| T2 [0.87, 0.93] | 209 (41.22%) | 1.07 (0.84, 1.36) | 0.599 |  | 1.59 (1.17, 2.16) | 0.003 |  | 1.60 (1.17, 2.20) | 0.003 |
| T3 [0.93, 1.65] | 240 (45.20%) | 1.25 (0.99, 1.59) | 0.060 |  | 1.78 (1.27, 2.48) | <0.001 |  | 1.70 (1.21, 2.40) | 0.002 |
| *P-value* for trend |  | 0.064 | |  | <0.001 | |  | 0.003 | |
| *Middle [84.00, 92.67]* |  |  |  |  |  |  |  |  |  |
| WHR |  |  |  |  |  |  |  |  |  |
| Per *SD* increase | 708 (42.17%) | 1.28 (1.15, 1.41) | <0.001 |  | 1.41 (1.21, 1.65) | <0.001 |  | 1.40 (1.19, 1.64) | <0.001 |
| Central obesity |  |  |  |  |  |  |  |  |  |
| No | 216 (37.18%) | *Ref* |  |  | *Ref* |  |  | *Ref* |  |
| Yes | 492 (44.81%) | 1.37 (1.12, 1.69) | 0.003 |  | 1.60 (1.18, 2.18) | 0.003 |  | 1.52 (1.11, 2.09) | 0.009 |
| WHR tertiles |  |  |  |  |  |  |  |  |  |
| T1 [0.59, 0.87] | 192 (36.29%) | *Ref* |  |  | *Ref* |  |  | *Ref* |  |
| T2 [0.87, 0.93] | 237 (40.03%) | 1.17 (0.92, 1.49) | 0.199 |  | 1.39 (1.01, 1.91) | 0.046 |  | 1.36 (0.98, 1.89) | 0.067 |
| T3 [0.93, 1.68] | 279 (50.00%) | 1.76 (1.38, 2.24) | <0.001 |  | 2.28 (1.58, 3.30) | <0.001 |  | 2.19 (1.50, 3.21) | <0.001 |
| *P-value* for trend |  | <0.001 | |  | <0.001 | |  | <0.001 | |
| *High [93.00, 129.67]* |  |  |  |  |  |  |  |  |  |
| WHR |  |  |  |  |  |  |  |  |  |
| Per *SD* increase | 825 (47.77%) | 1.04 (0.94, 1.15) | 0.399 |  | 1.38 (1.18, 1.63) | <0.001 |  | 1.33 (1.13, 1.58) | <0.001 |
| *P-value* for interaction |  | 0.004 | |  | 0.011 | |  | 0.005 | |
| Central obesity |  |  |  |  |  |  |  |  |  |
| No | 281 (45.47%) | *Ref* |  |  | *Ref* |  |  | *Ref* |  |
| Yes | 544 (49.05%) | 1.15 (0.95, 1.41) | 0.153 |  | 1.70 (1.27, 2.28) | <0.001 |  | 1.60 (1.18, 2.15) | 0.002 |
| *P-value* for interaction |  | 0.457 | |  | 0.889 | |  | 0.731 | |
| WHR tertiles |  |  |  |  |  |  |  |  |  |
| T1 [0.70, 0.87] | 265 (48.27%) | *Ref* |  |  | *Ref* |  |  | *Ref* |  |
| T2 [0.87, 0.93] | 270 (46.71%) | 0.94 (0.74, 1.19) | 0.601 |  | 1.28 (0.94, 1.75) | 0.122 |  | 1.19 (0.86, 1.63) | 0.294 |
| T3 [0.93, 1.35] | 290 (48.33%) | 1.00 (0.80, 1.26) | 0.983 |  | 1.70 (1.19, 2.44) | 0.004 |  | 1.55 (1.07, 2.23) | 0.020 |
| *P-value* for trend |  | 0.983 | |  | 0.003 | |  | 0.019 | |
| *P-value* for interaction |  | 0.025 | |  | 0.153 | |  | 0.063 | |
| Abbreviations: DBP, diastolic blood pressure; WHR, waist hip rate; *Ref*, reference; OR, odds ratio; CI, confidence interval; *SD*, standard deviation. ModelⅠadjusted for age, sex, SBP, DBP, HR and BMI. ModelⅡadjusted forage, sex, SBP, DBP, HR, BMI, smoking status, ALT, HDL-C, eGFR, DM, CHD, history of stroke and antihypertensive medications. | | | | | | | | | |

| **Supplementary Table 10. Effect size of BMI tertiles on baPWV levels in prespecified and exploratory subgroups** | | | | | | | | |
| --- | --- | --- | --- | --- | --- | --- | --- | --- |
| **Subgroup** | **T1 [13.83, 21.68]** |  | **T2 [21.68, 24.69]** | |  | **T3 [24.69, 46.43]** | | **Interactive *P-value*** |
|  |  |  | ***β* (95%CI)** | ***P-value*** |  | ***β* (95%CI)** | ***P-value*** |  |
| Age tertiles (years) |  |  |  |  |  |  |  | 0.191 |
| Low [29.0, 60.0] | *Ref* |  | -0.14 (-0.48, 0.20) | 0.423 |  | -0.73 (-1.10, -0.36) | <0.001 |  |
| Middle [61.0, 68.0] | *Ref* |  | -0.60 (-0.94, -0.25) | <0.001 |  | -0.94 (-1.34, -0.55) | <0.001 |  |
| High [69.0, 93.0] | *Ref* |  | -0.79 (-1.22, -0.37) | <0.001 |  | -1.31 (-1.82, -0.80) | <0.001 |  |
| Sex |  |  |  |  |  |  |  | 0.071 |
| Male | *Ref* |  | -0.59 (-0.90, -0.28) | <0.001 |  | -0.79 (-1.15, -0.42) | <0.001 |  |
| Female | *Ref* |  | -0.61 (-0.92, -0.30) | <0.001 |  | -1.27 (-1.61, -0.93) | <0.001 |  |
| SBP (mmHg) |  |  |  |  |  |  |  | 0.281 |
| <140 | *Ref* |  | -0.20 (-0.52, 0.12) | 0.221 |  | -0.52 (-0.88, -0.16) | 0.004 |  |
| ≥140 | *Ref* |  | -0.79 (-1.08, -0.51) | <0.001 |  | -1.33 (-1.66, -1.00) | <0.001 |  |
| DBP (mmHg) |  |  |  |  |  |  |  | 0.403 |
| <90 | *Ref* |  | -0.51 (-0.77, -0.24) | <0.001 |  | -0.88 (-1.19, -0.57) | <0.001 |  |
| ≥90 | *Ref* |  | -0.71 (-1.06, -0.35) | <0.001 |  | -1.25 (-1.65, -0.85) | <0.001 |  |
| Central obesity |  |  |  |  |  |  |  | 0.876 |
| No | *Ref* |  | -0.55 (-0.87, -0.23) | <0.001 |  | -0.92 (-1.43, -0.42) | <0.001 |  |
| Yes | *Ref* |  | -0.65 (-0.95, -0.35) | <0.001 |  | -1.05 (-1.35, -0.75) | <0.001 |  |
| DM |  |  |  |  |  |  |  | 0.668 |
| No | *Ref* |  | -0.64 (-0.88, -0.41) | <0.001 |  | -1.05 (-1.33, -0.78) | <0.001 |  |
| Yes | *Ref* |  | -0.44 (-1.02, 0.14) | 0.135 |  | -1.06 (-1.69, -0.43) | 0.001 |  |
| eGFR (ml/min/1.73m^^2^) |  |  |  |  |  |  |  | 0.596 |
| ≥60.0 | *Ref* |  | -0.30 (-1.10, 0.49) | 0.454 |  | -0.92 (-1.82, -0.03) | 0.043 |  |
| <60.0 | *Ref* |  | -0.65 (-0.87, -0.43) | <0.001 |  | -1.08 (-1.34, -0.82) | <0.001 |  |
| Antihypertensive medications |  |  |  |  |  |  |  | 0.732 |
| No | *Ref* |  | -0.72 (-1.06, -0.38) | <0.001 |  | -1.21 (-1.62, -0.80) | <0.001 |  |
| Yes | *Ref* |  | -0.56 (-0.84, -0.28) | <0.001 |  | -0.99 (-1.31, -0.68) | <0.001 |  |
| Abbreviations: BMI, body mass index (kg/m^2); baPWV, brachial-ankle pulse wave velocity; SBP, systolic blood pressure; DBP, diastolic blood pressure; DM, diabetes mellitus; eGFR, estimated glomerular filtration rate; *Ref*, reference; *β*, effect size; CI, confidence interval.  Each stratification adjusted for age, sex, SBP, DBP, HR, WHR, eGFR, DM, CHD, history of stroke and antihypertensive medications except the subgroup variable. | | | | | | | | |
|  |  |  |  |  |  |  |  |  |
