# Supplementary figures and images for: Does body mass index or waist-hip ratio correlate with arterial stiffness based on brachial-ankle pulse wave velocity in Chinese rural adults with hypertension?
Source: BMC Cardiovasc Disord. 2021 Dec 1;21:573. doi: 10.1186/s12872-021-02390-y (PMC8638469; doi:10.1186/s12872-021-02390-y)

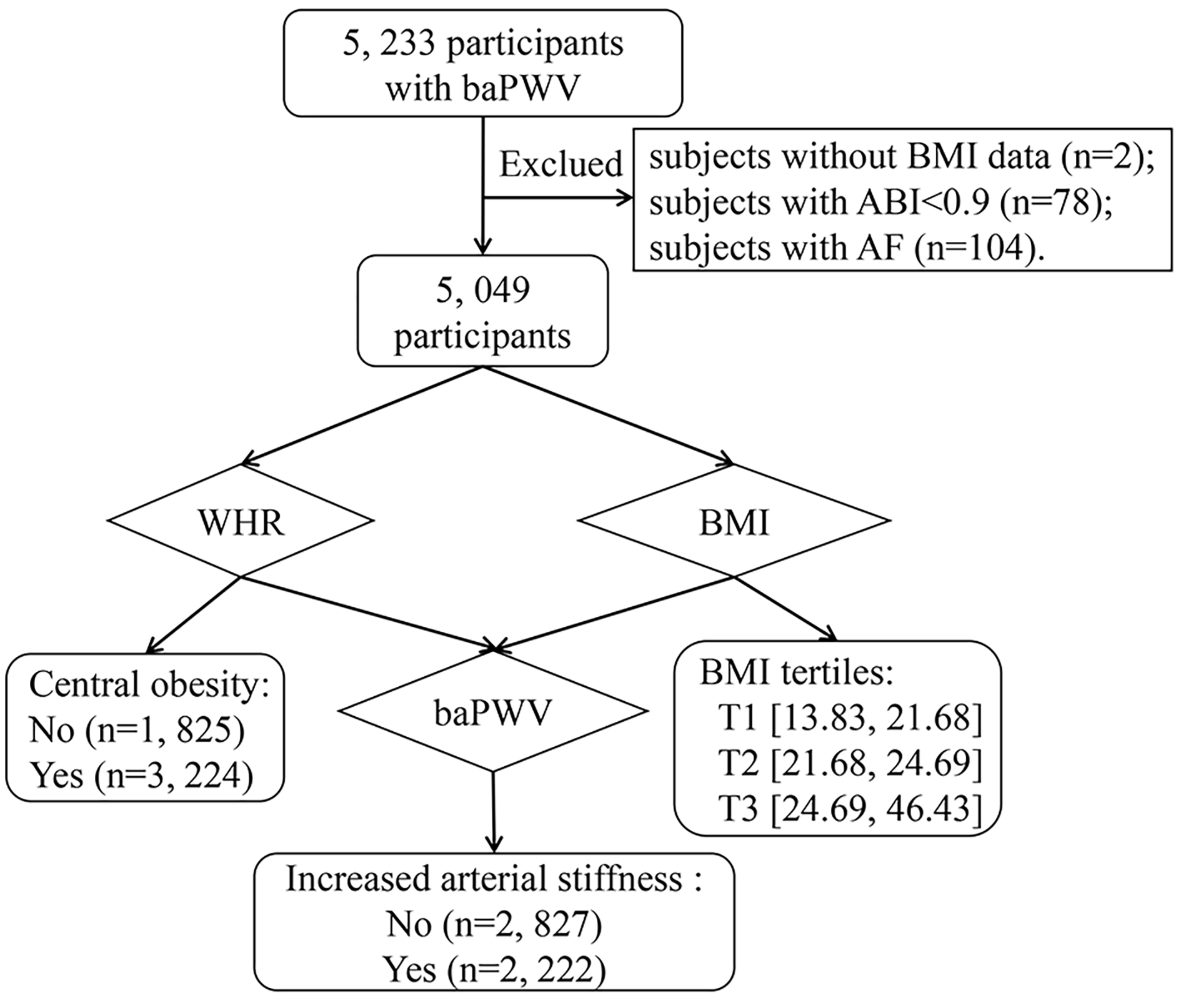

Supplement: Supplementary file 2 — Additional file 2: Fig. 1 The data flow chart of participants in our analysis. baPWV, brachial-ankle pulse wave velocity; BMI, body mass index; ABI, ankle brachial index; AF, atrial fibrillation; WHR, waist hip rate. [file 12872_2021_2390_MOESM2_ESM.tif]

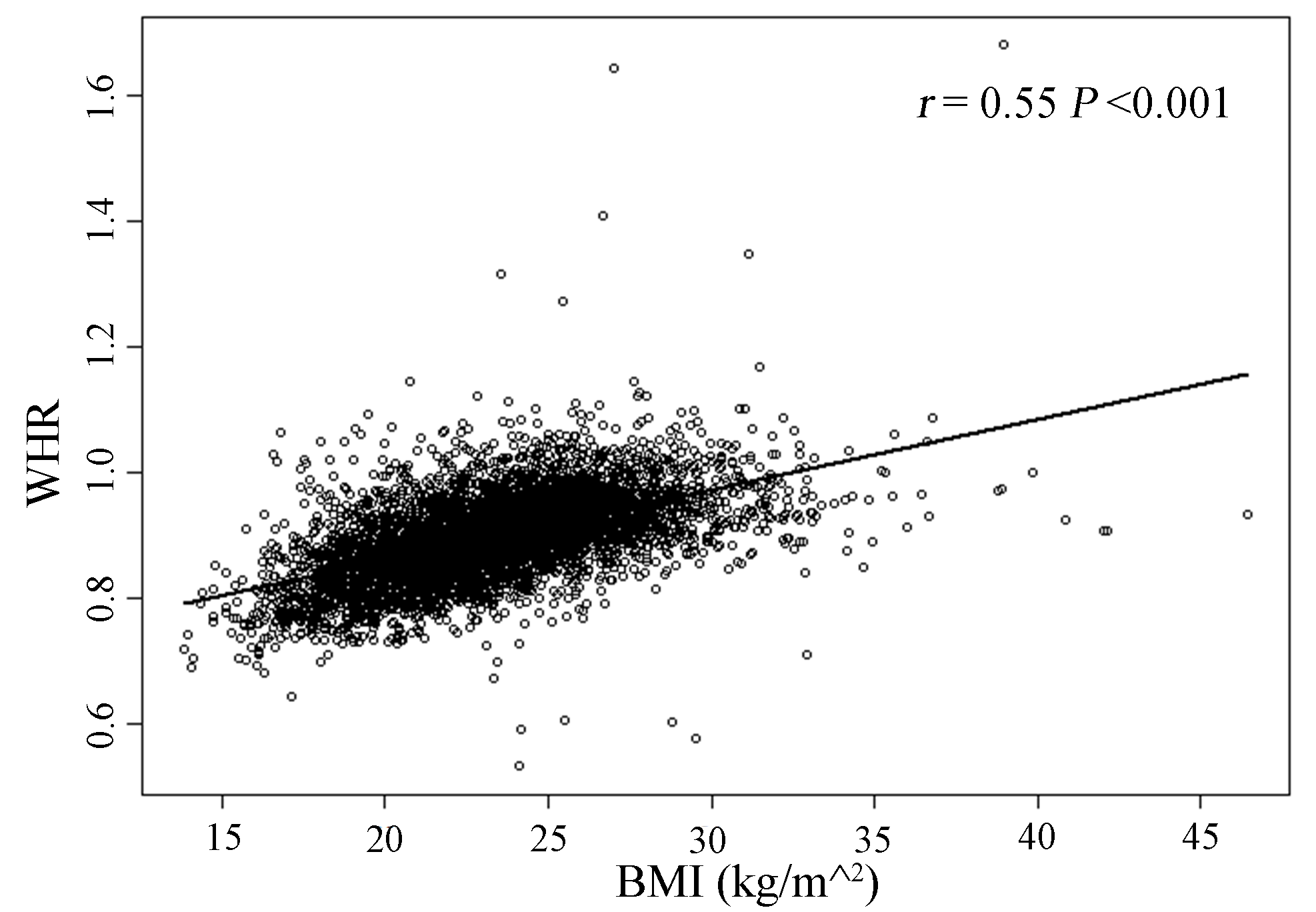

Supplement: Supplementary file 3 — Additional file 3: Fig. 2 The correlation between BMI levels and WHR levels. [file 12872_2021_2390_MOESM3_ESM.tif]
